# Effect of exercise training on the renin–angiotensin–aldosterone system: a meta–analysis

**DOI:** 10.1038/s41371-023-00872-4

**Published:** 2023-11-28

**Authors:** Biggie Baffour-Awuah, Melody Man, Karla F. Goessler, Véronique A. Cornelissen, Gudrun Dieberg, Neil A. Smart, Melissa J. Pearson

**Affiliations:** 1 Clinical Exercise Physiology, School of Science and Technology, Faculty of Science, Agriculture, Business and Law, University of New England, Armidale, NSW 2351 Australia; 2 Applied Physiology & Nutrition Research Group, School of Physical Education and Sport, University of São Paulo, São Paulo, Brazil; 3 Cardiovascular Exercise Physiology Unit, Department of Rehabilitation Sciences, KU Leuven, Leuven, Belgium

**Keywords:** Hypertension, Lifestyle modification

## Abstract

Blood pressure (BP) management reduces the risk of cardiovascular disease (CVD). The renin–angiotensin–aldosterone system (RAAS) plays an important role in regulating and maintaining blood volume and pressure. This analysis aimed to investigate the effect of exercise training on plasma renin, angiotensin-II and aldosterone, epinephrine, norepinephrine, urinary sodium and potassium, BP and heart rate (HR). We systematically searched PubMed, Web of Science, and the Cochrane Library of Controlled Trials until 30 November 2022. The search strategy included RAAS key words in combination with exercise training terms and medical subject headings. Manual searching of reference lists from systematic reviews and eligible studies completed the search. A random effects meta-analysis model was used. Eighteen trials with a total of 803 participants were included. After exercise training, plasma angiotensin-II (SMD −0.71; 95% CI −1.24, −0.19; *p* = 0.008; *n* = 9 trials), aldosterone (SMD −0.37; 95% CI −0.65, −0.09; *p* = 0.009; *n* = 8 trials) and norepinephrine (SMD −0.82; 95% CI −1.18, −0.46; *p* < 0.001; *n* = 8 trials) were reduced. However, plasma renin activity, epinephrine, and 24-h urinary sodium and potassium excretion remained unchanged with exercise training. Systolic BP was reduced (MD −6.2 mmHg; 95% CI −9.9, −2.6; *p* = 0.001) as was diastolic BP (MD −4.5 mmHg; 95% CI −6.9, −2.1; *p* < 0.001) but not HR (MD −3.0 bpm; 95% CI −6.0, 0.4; *p* = 0.053). Exercise training may reduce some aspects of RAAS and sympathetic nervous system activity, and this explains some of the anti-hypertensive response.

## Introduction

Hypertension is a major independent and preventable risk factor of cardiovascular disease (CVD) [1] and physical inactivity contributes to hypertension [2, 3]. Engaging in regular exercise is known to reduce blood pressure (BP) [4, 5] and stimulates physiological adaptations for general health improvements [6]. However, the reduction in BP following exercise training is regulated by multifaceted mechanisms which remain elusive [5, 7]. The renin–angiotensin–aldosterone system (RAAS) is an important regulator of BP [8]. Renin is secreted by the kidney in response to low arterial pressure, sympathetic activation, sodium deficiency or dehydration. Renin converts angiotensinogen to angiotensin-I which is then converted to angiotensin-II by angiotensin-converting enzyme (ACE) [9, 10]. Angiotensin-II is the major mediator of RAAS causing aldosterone release, vasoconstriction and fluid and electrolyte retention to increase blood volume and BP [11–13]. Increased RAAS activation may lead to the development of hypertension due to several environmental and physiological factors (e.g. vasoconstriction, fluid and electrolyte imbalances) [14–17]. Exercise training may exert a counter regulatory effect on the RAAS that initiates vasodilation and cardio-protection, by suppressing angiotensin-II release. A previous meta-analysis has shown that chronic endurance training reduced BP which the authors attributed to reductions in systemic vascular resistance via RAAS and sympathetic nervous system (SNS) mediation [5]. Goessler et al. [18] in a meta-analysis of 11 randomised controlled trials (RCTs) of healthy individuals showed a reduction in plasma renin activity but unchanged angiotensin-II and aldosterone levels. Since the systematic review by Goessler et al., there have been 9 new trials conducted in different clinical populations that now warrant further analysis of the effects of exercise training on the RAAS. The aim of this meta-analysis was to investigate the effect of exercise training on plasma renin activity, angiotensin-II, aldosterone, epinephrine, norepinephrine, urinary sodium, potassium, BP and heart rate (HR). We hypothesised that exercise training would reduce RAAS parameters and SNS activity, and ultimately reduce BP and resting HR. In addition, sub-analyses aimed to explore the effects of health status, medication use, and exercise modality.

## Materials and methods

This meta-analysis was registered with the International Prospective Register of Systematic Reviews (PROSPERO) [CRD42021255225] [19]. The review protocol can be accessed at PROSPERO website.

### Search strategy

We conducted a systematic literature search in PubMed, Web of Science and the Cochrane Library of Controlled Trials up until 30 November 2022. The search strategy included RAAS key words and in combination with exercise training and MeSH terms. This was supplemented by manually searching reference lists from systematic reviews and eligible studies for additional studies. The strategy for the database searches is documented in the online resources (Supplementary Table S1).

### Inclusion and exclusion criteria

We included RCTs in adults (over 18 years) that assessed RAAS parameters after exposing participants to different types of exercise training (e.g. aerobic, resistance or combined) as the main intervention for a minimum of 4 weeks duration. Crossover studies were excluded if the washout period was less than two weeks. Identified studies that did not report any of the required outcomes were excluded.

Two authors (BB, MM) assessed all identified articles independently for eligibility, and consulted two reviewers (MJP, GD) for any disagreement to be resolved.

### Comparisons

Included studies compared exercise training intervention group(s) to a non-exposed control (usual care) group or a sham intervention (supervised stretching/callisthenics) group.

### Outcome measures

The primary outcome measures were changes in plasma renin activity, angiotensin-II and aldosterone. The secondary outcome measures were change in epinephrine, norepinephrine, urinary sodium and potassium excretion, BP and HR.

Please note that some of the outcome measures mentioned in the initial protocol might not be feasible if the reporting of these is limited and insufficient for meta-analysis. In addition to the protocol, we did include BP and HR measurements as additional outcome measures as these are relevant for a RAAS study.

### Data extraction

From each included study we extracted the first author’s name, year of publication, country, study design, type of study population, participants’ baseline characteristics (including age, gender, number of participants, and resting BP). In addition, the characteristics of training interventions (i.e. exercise programs, intensity, duration and frequency of the protocol) and the changes in the desired outcome variables were obtained. Data extraction was conducted independently by two authors (BB, MM) using a predesigned data extraction sheet. Reviewers (GD, MJP, NAS) were consulted for resolution of any disagreements. For outcome data reported in figures only, the WebPlotDigitizer (version 4.5) [20] computer software was used to extract the relevant dataset.

### Statistical analyses

Data sets were organised and descriptive analyses performed using Excel 2016 for all included studies. Meta-analyses were completed in Comprehensive Meta-Analysis (CMA) V4 (Biostat Inc, NJ, USA). Where outcome variables were assessed by different methods and reported in different units of measure, the standardised mean difference (SMD) for the outcome measures studied were pooled using a random effects model (DerSimonian-Laird). For two included studies with multiple exercise intervention groups [21, 22], data was combined for the effect size for the parameter measured using ReviewManager 5.4 software (The Nordic Cochrane Centre, Copenhagen, Denmark). Forest plots were generated using CMA to provide visual representation of the effect sizes. Sensitivity analyses were conducted using ‘one study removed’ statistics in CMA for overall assessment of the intervention effect. Sub-analyses were performed on RAAS outcome parameters that were statistically significant and systolic/diastolic BP by health status, medication use, exercise modality and control activity. The level of statistical significance was set at *p* < 0.05.

### Heterogeneity and publication bias

Statistical heterogeneity (*I*^2^) was assessed for inconsistency among studies with the values of 25%, 50%, and 75% representing low, moderate, and high degrees of heterogeneity [23]. Publication bias was evaluated by visual inspection of the funnel plot for all outcomes with Egger’s regression test [24].

### Quality of study assessment

The validated Tool for the assEssment of Study qualiTy and reporting in EXercise (TESTEX) [25] was used to assess the methodological quality of the included studies. This is a 15-point assessment criteria (5 points for study quality and 10 points for reporting) designed specifically for use in exercise training studies.

## Results

**Fig. 1:**
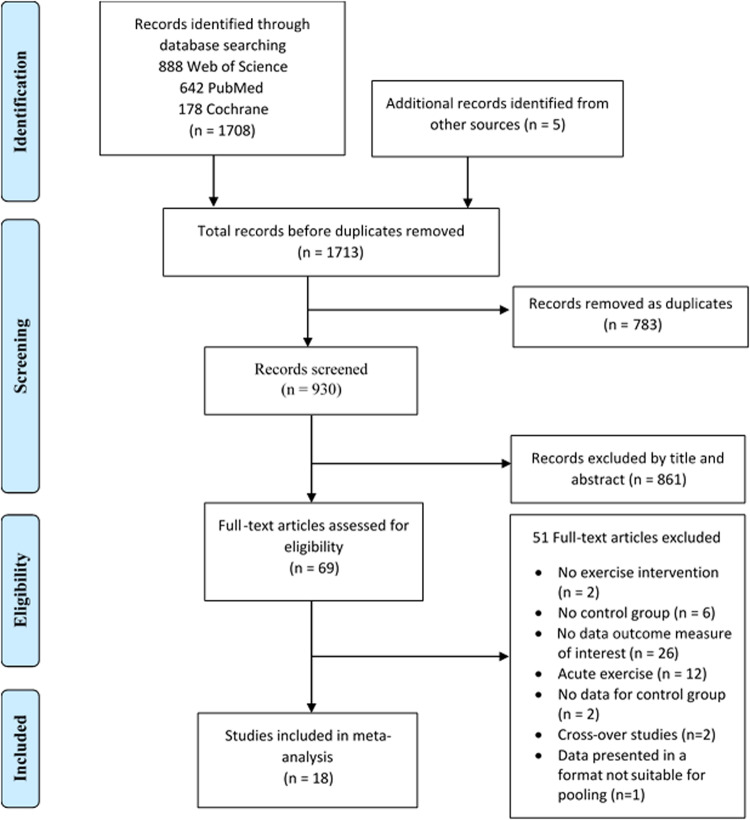
PRISMA flow diagram of included studies.

The initial searches identified a total of 1708 records plus an additional 5 records through manual searching, 783 duplicate records were removed leaving 930 records for screening. A total of 861 records were excluded after screening titles and abstracts. The remaining 69 full-text articles were assessed for eligibility. A further 51 studies were excluded with reasons provided in the PRISMA flow diagram (Fig. 1). Eighteen studies were included in the quality synthesis and meta-analysis.

### Study characteristics

Of the 18 RCTs [21, 22, 26–41] included in this review, most of the studies [21, 22, 26, 29–31, 39] were conducted in the USA (7 studies), Japan had 4 studies [33, 34, 36, 37], Brazil had three studies [32, 38, 40], with one study each in China [41], Italy [35], Poland [28] and Turkey [27]; studies were published between 1987 and 2022. The studies included a total of 803 randomised participants (exercise and control group, *n* = 458 and 349, respectively). Most studies included both male and female participants except for three trials which included only male [28] or female [27, 39] participants. The median age was 56.7 (range: 47–70.4) years. This review included trials in healthy adults [21, 26, 31, 39], participants with hypertension [22, 32–34, 36–38, 40, 41] and pre-hypertension [27], as well as patients with heart failure [29, 30, 35], and coronary artery disease [28]. At baseline, the average resting systolic BP was 140.2 mmHg (range 112–162.2 mmHg) and diastolic BP was 84.6 mmHg (range 65–102 mmHg). In 9 trials, participants taking medications either continued their medication [22, 28–30, 32, 35, 40] or discontinued [36–38] it before the start of the intervention. The duration of the trial interventions varied from 4 to 37 weeks and the frequency ranged from 3 to 7 training sessions per week. The type of exercise adopted by trials included isolated aerobic training in 12 trials [22, 27–30, 33–39], resistance training in two trial [26, 40] and two trials employed a combination of aerobic and resistance training [21, 31]. In two trials, participants performed water-based calisthenics and walking [32], and tai chi [41]. The majority of the trials used ‘no exercise’ as a control intervention except in 3 trials where supervised stretching [22, 26] and isometric callisthenics (posture and breathing techniques) [38] were performed to control for placebo effect. The characteristics for included studies are detailed in Table 1.

**Table 1:** Characteristics of included randomised controlled trials.

Study /country	Study population / Age (years)	Mean resting BP (mmHg)	Outcome measures	Protocol – Duration and frequency per week	
				Exercise intervention group (EIG)	Control (Con)
Anton et al. [26] USA	26 (7 M, 19 F) healthy adults EIG: 13 (3 M, 10 F) / 52 ± 2 Con: 13 (4 M, 9 F) / 53 ± 2		BP, body composition, plasma concentrations of norepinephrine, endothelin-1, and angiotensin-II, blood glucose, lipids, lipoproteins, basal femoral blood flow, femoral artery lumen diameter, femoral artery IMT and MBV, and CO	13 weeks of supervised resistance (seated chest press, bilateral leg press, upper back, hamstrings, shoulders, triceps, biceps, calves, and abdominal) training, 12 reps/set at 75% 1-RM with 2 min rest interval, 3 sessions per week	13 weeks of 2 supervised and one home-based stretching exercise sessions per week
Azadpour et al. [27] Turkey	24 obese prehypertensive postmenopausal women EIG: 12 / 57.6 ± 4.3 Con: 12 / 56.7 ± 4.2	EIG SBP: 127.92 ± 4.74 DBP: 82.00 ± 1.54 Con SBP: 129.58 ± 3.85 DBP: 82.33 ± 1.30	BP, HR, body composition, VO_2max_, angiotensin-converting enzyme and β2-adrenergic receptor gene expression in leucocytes, plasma angiotensin-II, and flow-mediated dilation	10 weeks of 25–40 min per day moderate-intensity aerobic exercise training (treadmill exercise) progressively at 50%–70% HR reserve, 3 times per week	No intervention
Bilińska et al. [28] Poland	100 coronary artery disease male patients 56 ± 6 EIG: 50 / 57 ± 6 Con: 50 / 56 ± 6	EIG SBP: 117.39 ± 8.15 DBP: 71 ± 2.53 Con SBP: 117.39 ± 3.61 DBP: 70.71 ± 2.53	BP, HR, VO_2max_, HR recovery, HR variability, blood glucose, lipids, CO, SV, TPR, plasma renin activity, adrenaline, noradrenaline and atrial natriuretic peptide	6 weeks of 60 min aerobic training (4 min × 2 min rest bicycle ergometer exercise) at 70–80% HR_max,_ 3 times per week	No intervention
Braith et al. [29] USA	19 heart failure patients EIG: 10 / 61 ± 6 Con: 9 / 62 ± 7		Angiotensin-II, aldosterone, vasopressin, and atrial natriuretic peptide	16 weeks aerobic training (treadmill exercise) at 40–80% VO_2max_ progressively for a duration of 10–45 min, as tolerated, 3 times per week	No intervention
Brubaker et al. [30] USA	59 (39 M, 20 F) older heart failure patients 70.2 ± 5.1 EIG: 30 (23 analysed) / 70.4 ± 5.3 Con: 29 (21 analysed) / 69.9 ± 6.3	EIG SBP: 133.3 ± 19.4 DBP: 77.1 ± 9.8 Con SBP: 140.9 ± 24.1 DBP: 77.9 ± 10.4	Left ventricular structure and function, Health-Related Quality of Life (HRQOL), C-terminal atrial natriuretic peptide and brain natriuretic peptide-32, angiotensin-II, aldosterone, norepinephrine, and VO_2max_	16 weeks aerobic training (walking and cycle ergometry) at 40–70% of HR reserve progressively for a duration of 60–80 min, 3 times per week	No intervention
Carroll et al. [31] USA	38 (16 M, 22 F) healthy older adults 60–82 EIG: 29 / 68.4 ± 5.2 Con: 9 / 66.6 ± 6.4	EIG SBP: 125 ± 16 DBP: 76 ± 6 Con SBP: 122 ± 10 DBP: 80 ± 8	BP, plasma concentrations of adrenocorticotropic hormone, vasopressin, norepinephrine, epinephrine, aldosterone, sodium, potassium, protein, haemoglobin, and haematocrit	26 weeks aerobic training (treadmill walking, or stairclimbing or either of activities plus resistance) at 75–84% of HR reserve progressively for a duration of 30–45 min, as tolerated, 3 times per week	No intervention
Correa et al. [40] Brazil	90 hypertensive patients 58 ± 9 EIG_1_: 30 (18 M, 12 F) / 58 ± 9 EIG_2_: 30 (20 M, 10 F) / 60 ± 8 Con: 30 (19 M, 11 F) / 57 ± 6	EIG_1_ SBP: 145 ± 3 DBP: 90 ± 5 EIG_2_ SBP: 143 ± 6 DBP: 91 ± 4 Con SBP: 144 ± 5 DBP: 90 ± 2	BP, HR, body composition, angiotensin-converting enzyme, angiotensin-II, vasopressin, bradykinin, and redox balance	EIG_1_ – 24 weeks of resistance training (bench press, leg press at 45◦, seated row, leg extension, shoulder press, leg curl, barbell biceps curl, and triceps pulley) which consisted of 3 two-month mesocycles periodisation performed 3 sessions per week progressively at increasing intensities of 50, 60 and 70% 1-RM with 3 sets of decreasing repetition of 12, 10 and 8 respectively for first, second and third mesocycles. EIG_2_ – 24 weeks of resistance training as in EIG_1,_ but reduced intensities at 30, 40 and 50% 1-RM respectively for first, second and third mesocycles plus moderate blood flow restriction	No intervention
Cortez-Cooper et al. [21] USA	37 healthy sedentary adults 52 ± 2 EIG_1_: 13 (3 M, 10 F) / 52 ± 2 EIG_2_: 12 (3 M, 9 F) / 51 ± 1 Con: 12 (4 M, 8 F) / 54 ± 2	EIG_1_ SBP: 113 ± 3 DBP: 66 ± 2 EIG_2_ SBP: 118 ± 3 DBP: 68 ± 2 Con SBP: 122 ± 4 DBP: 66 ± 3	BP, HR, body composition, VO_2max_, arterial stiffness and compliance, blood glucose, lipids, endothelin-1, and angiotensin-II	EIG_1_ – 13 weeks of supervised strength (seated chest press, horizontal leg press, shoulder press, abdominal crunches, seated hamstring curls, seated row, seated calf raises, low back extension, tricep curls, and bicep dumbbell curls) training; 70% 1-RM; 1 set; 12 reps/set, 3 days per week EIG_2_ – 13 weeks of strength training as in EIG_1,_ 2 days per week plus aerobic exercise (either walking or cycling at 60–75% HR reserve) on separate days, 30–45 min per session, 2 days per week	13 weeks of supervised stretching exercise 3 times per week
Cruz et al. [32] Brazil	44 (23 M, 21 F) resistant hypertensive patients 53.3 ± 0.9 EIG: 28 (14 M, 14 F) / 54.4 ± 1.2 Con: 16 (9 M, 7 F) / 52.4 ± 1.5	EIG SBP: 162.2 ± 23.2 DBP: 83.8 ± 2.5 Con SBP: 157.6 ± 17.6 DBP: 86.4 ± 2.5	BP, HR, VO_2max_, plasma concentrations of nitric oxide, endothelin-1, aldosterone, renin, norepinephrine, epinephrine, and endothelial function	12 weeks of 60 min callisthenic exercises against water resistance and walking in a heated pool; 3 times per week	No intervention
Hagberg et al. [22] USA	30 hypertensive patients 64 ± 3 EIG_1_: 11 / 52 ± 2 EIG_2_: 10 / 51 ± 1 Con: 9 / 54 ± 2	EIG_1_ SBP: 158 ± 18 DBP: 90 ± 10 EIG_2_ SBP: 160 ± 21 DBP: 100 ± 10 Con SBP: 152 ± 9 DBP: 90 ± 7	BP, HR, VO_2max_, body weight, percentage body fat, SV, CO, TPR, blood volume, plasma volume, plasma renin, urinary sodium, and haematocrit	37 weeks of supervised aerobic training, 3 times per week EIG_1_ – 51 min low intensity home-based walking at 53% VO_2max_ or EIG_2_ – 51 min moderate-intensity walking, jogging, cycle ergometry, and treadmill walking at 73% VO_2max_ per session progressively	No intervention
Higashi et al. [34] Japan	27 hypertensive patients EIG: 20 (14 M, 6 F) / 53 ± 10 Con: 7 (6 M, 1 F) / 51 ± 8	EIG SBP: 155 ± 6.6 DBP: 96 ± 4.9 Con SBP: 155.4 ± 8.3 DBP: 97.6 ± 4.3	BP, HR, body weight, basal forearm blood flow, forearm blood flow and vascular resistance, lipids, norepinephrine, renin activity, serum glucose, insulin, urinary sodium and potassium	12 weeks of 30 min brisk walking at 52% VO_2max_, 5–7 times per week	No intervention
Higashi et al. [33] Japan	17 hypertensive patients 47 ± 10 EIG: 10 (7 M, 3 F) / 49 ± 10 Con: 7 (6 M, 1 F) / 44 ± 8	EIG SBP: 151.6 ± 7 DBP: 96.2 ± 4.7 Con SBP: 155.6 ± 8.9 DBP: 97.6 ± 4.8	BP, HR, body weight, basal forearm blood flow, forearm blood flow and vascular resistance, level of cholesterols and triglycerides, plasma norepinephrine and renin activity, aldosterone, nitric oxide, serum glucose and insulin, urinary sodium and potassium	12 weeks of 30 min brisk walking at 52% VO_2max_, 5–7 times per week	No intervention
Lin et al. [41] China	99 hypertensive patients EIG: 50 / 64.2 ± 4 Con: 49 / 63.8 ± 4.4	EIG SBP: 143.9 ± 7.1 DBP: 85.6 ± 5.4 Con SBP: 142.9 ± 9.3 DBP: 85 ± 5.5	BP, BMI, serum angiotensin-II and nitric oxide concentration	12 weeks of 30 min of tai chi of an exercise session total duration of 60 min, 3 times per week	No intervention
Passino et al. [35] Italy	85 (74 M, 11 F) heart failure patients EIG: 44 (39 M, 5 F) / 60 ± 2 Con: 16 (35 M, 6 F) / 61 ± 2		VO_2max_, B-type natriuretic peptide, amino-terminal pro-brain natriuretic peptide, plasma renin activity, aldosterone, and catecholamines	36 weeks of 30 min cycling per week at 65% VO_2max_ at least 3 times per week	No intervention
Sakai et al. [36] Japan	29 (5 M, 24 F) hypertensive patients EIG: 16 (3 M, 13 F) / 56 ± 2 Con: 13 (2 M, 11 F) / 52 ± 2	EIG SBP: 156 ± 2 DBP: 92 ± 2 Con SBP: 150 ± 3 DBP: 93 ± 2	BP, HR, SV, cardiac index, TPR index, plasma volume index, plasma renin activity, plasma norepinephrine, urinary catecholamines, sodium and potassium	4 weeks of 60 min cycling at 40–60% VO_2max_, 3 times per week	No intervention
Urata et al. [37] Japan	20 hypertensive patients 51.2 EIG: 10 (4 M, 6 F) / 51.4 ± 2.8 Con: 10 (4 M, 6 F) / 51.0 ± 2.9	EIG SBP: 156.3 ± 4 DBP: 102.8 ± 3.5 Con SBP: 154 ± 3.9 DBP: 98 ± 2.9	BP, HR, VO_2max_, body weight, SV, cardiac index, TPR, blood volume, plasma concentration of norepinephrine, epinephrine, renin activity, angiotensin-I, and serum aldosterone, angiotensin-converting enzyme activity, and electrolytes	10 weeks of 60 min cycling at 40–60% VO_2max_, 3 times per week	No intervention
Waib et al. [38] Brazil	79 (31 M, 48 F) hypertensive patients EIG: 55(25 M, 30 F) / 49 Con: 24 (6 M, 18 F) / 53		BP, BMI, VO_2max_, arterial compliance, forearm blood flow, insulin resistance, cortisol, plasma concentrations of renin, aldosterone, C-peptide, lipids and glucose, urinary metanephrine, creatinine and uric acid	12 weeks of 60 min aerobic (jogging on treadmill) training sessions at 50% to 70% VO_2max_, 3 times per week	12 weeks of 60 min isometric calisthenics with special attention to posture and breathing techniques, 3 times per week
Yoshizawa et al. [39] USA	55 healthy sedentary postmenopausal women EIG_1_: 12 / 57 ± 1 EIG_2_:15 / 56 ± 1 Con_1_: 13 / 59 ± 1 Con_2_: 15 / 57 ± 1	EIG_1_ SBP: 120 ± 3 DBP: 74 ± 3 EIG_2_ SBP: 118 ± 3 DBP: 73 ± 2 Con_1_ SBP: 114 ± 4 DBP: 72 ± 3 Con_2_ SBP: 122 ± 6 DBP: 75 ± 3	BP, HR, BMI, VO_2max_, carotid arterial compliance, lipids, and angiotensin-II	8 weeks of 25–45 min aerobic exercise (walking or cycling) training, 3–5 days per week (2 supervised and additional home-based trainings) progressively at 60–75% HR_max_ for both exercise groups EIG_1_ – receive placebo + exercised an average 45 min and 4 days per week EIG_1_ – received lactotripeptides + exercised an average 45 min 4 days per week	8 weeks of daily dose of either placebo or lactotripeptides ingestion Con_1_ – received placebo Con_2_ – received lactotripeptides

*BMI* body mass index, *BP* blood pressure, *CO* cardiac output, *Con* control, *DBP* diastolic blood pressure, *EIG* exercise intervention group, *F* female, *HR* heart rate, *HR*_*max*_ heart rate maximum, *IMT* intima-media thickness, *M* male, *MBV* mean blood velocity, *Min* minute, *reps* repetitions, *RM* repetition maximum, *SBP* systolic blood pressure, *SV* stroke volume, *TPR* total peripheral resistance, *VO*_*2max*_ maximal oxygen consumption

### Effect of exercise training on the renin–angiotensin–aldosterone system

**Fig. 2:**
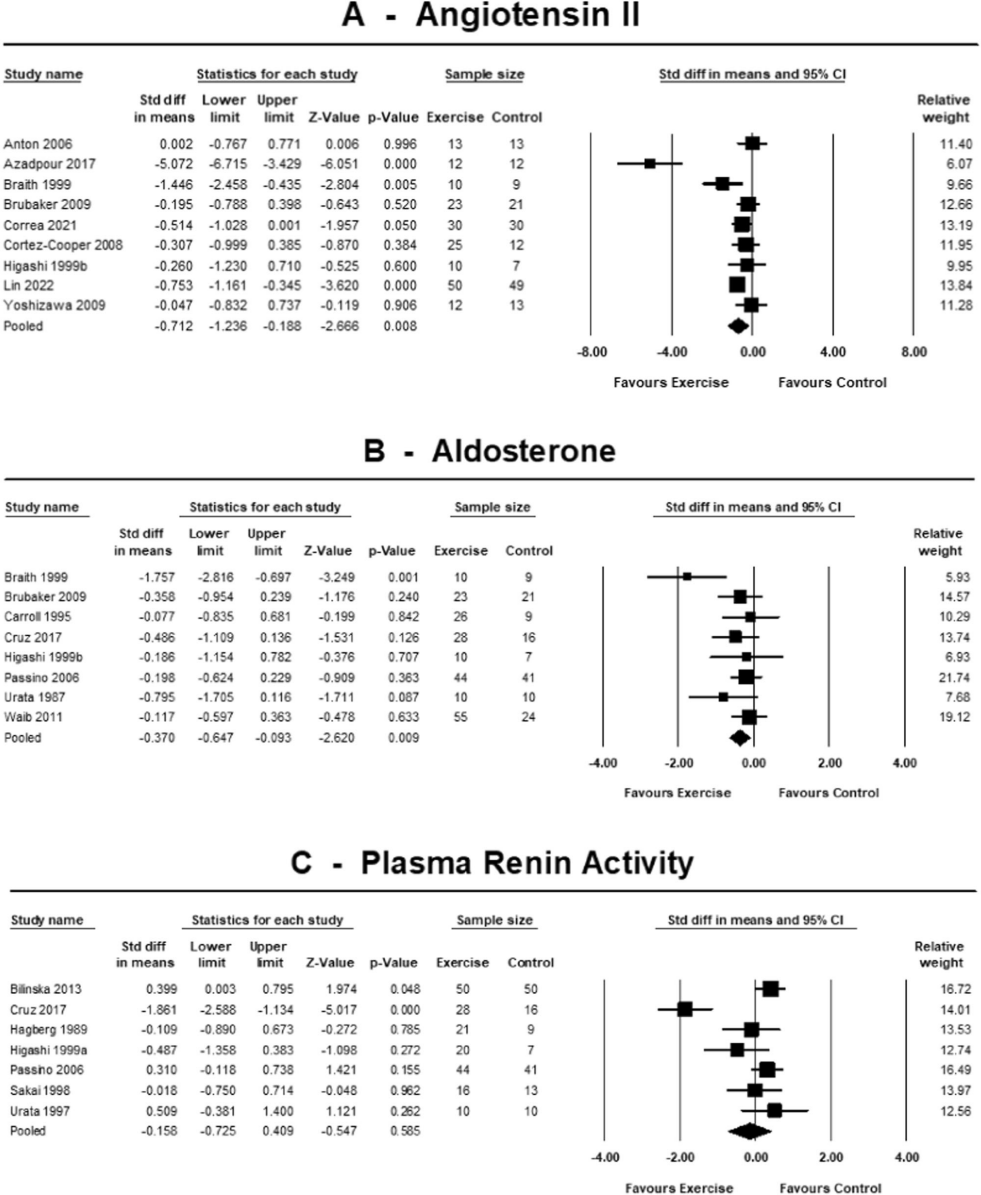
Standardised change in means of RAAS parameters. Forest plots showing the effects of exercise training on angiotensin-II [pg/mL] (**A**), aldosterone [pg/mL] (**B**) and plasma renin activity [ng/mL/h] (**C**) compared with control. A *p*-value < 0.05 represents a significant pooled standardised difference in means of overall effect. Horizontal lines across each present 95% CI for each study. The diamond represents the 95% CI for pooled estimates of effect of standardised mean difference.

Nine out of 18 included trials measured angiotensin-II plasma concentration. Exercise training decreased angiotensin-II concentration with SMD −0.71 (95% CI −1.24 to −0.19, *p* = 0.008; *I*^2^ = 79.4%, *p* < 0.001). Likewise, for the 8 trials measuring aldosterone concentration, pooled analysis showed a reduced SMD −0.37 (95% CI −0.65 to −0.09, *p* = 0.009; *I*^2^ = 28.9%, *p* = 0.197). However, plasma renin activity (measured in 7 trials) remained unchanged with SMD −0.16 (95% CI −0.73 to 0.41, *p* = 0.585; *I*^2^ = 82.1%, *p* < 0.001). The results of the training effect on RAAS parameters are displayed in Fig. 2.

### Effect of exercise training on sympathetic nervous system activity and electrolytes

**Fig. 3:**
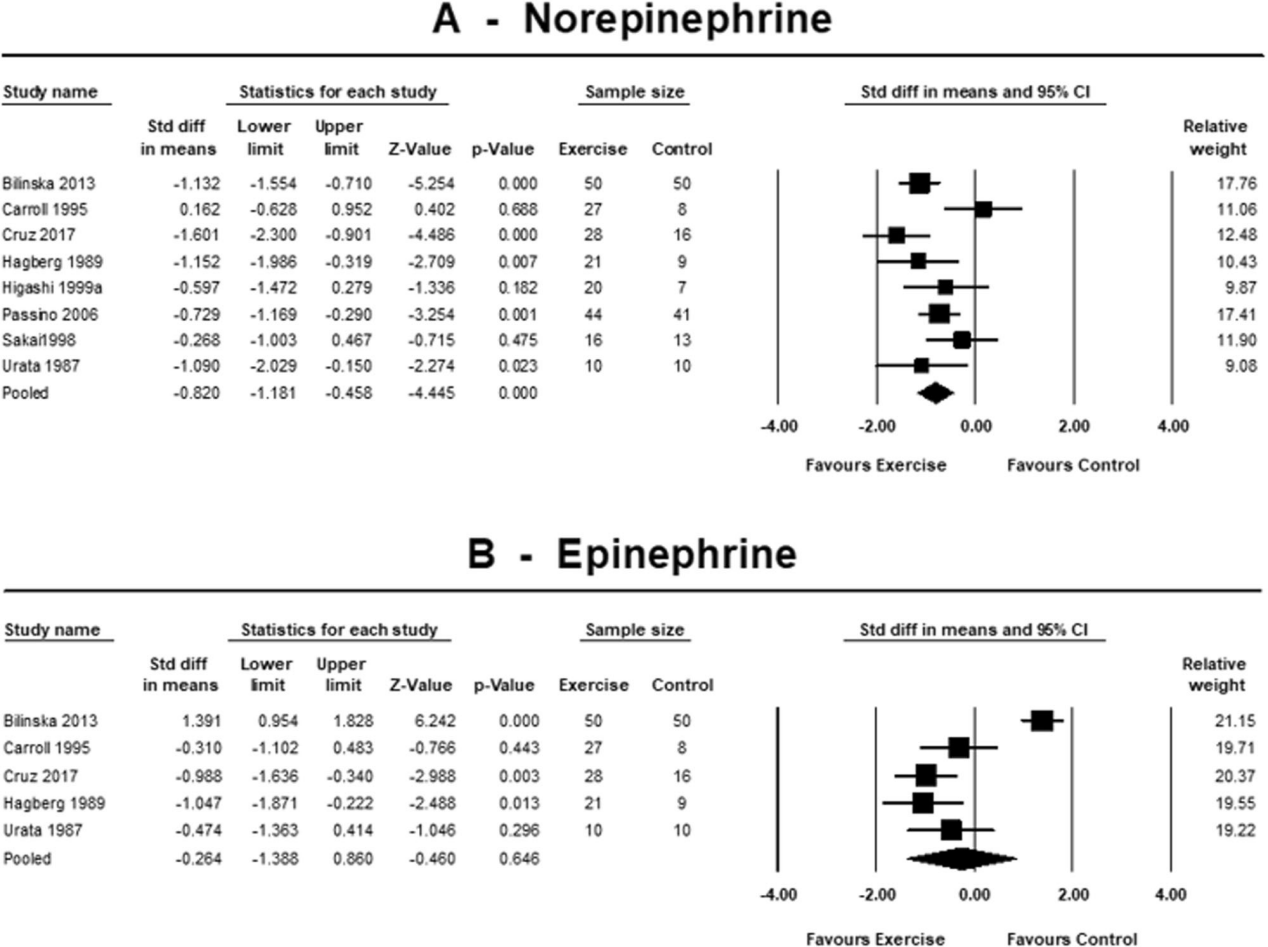
Standardised change in means of sympathetic nervous system activity. Forest plots showing the effects of exercise training on norepinephrine [pg/mL] (**A**) and epinephrine [pg/mL] (**B**) compared with control. A *p*-value < 0.05 represents a significant pooled standardised difference in means of overall effect. Horizontal lines across each present 95% CI for each study. The diamond represents the 95% CI for pooled estimates of effect of standardised mean difference.

Sympathetic nervous system activity was assessed by plasma norepinephrine and epinephrine levels (Fig. 3). In 8 trials, exercise training reduced norepinephrine with SMD −0.82 (95% CI −1.18 to −0.46, *p* < 0.001; *I*^2^ = 56.8%, *p* = 0.023). Five trials reported exercise training effect on epinephrine, but there was no change; SMD −0.26 (95% CI −1.39 to 0.860, *p* = 0.646; *I*^2^ = 92.6%, *p* < 0.001).

**Fig. 4:**
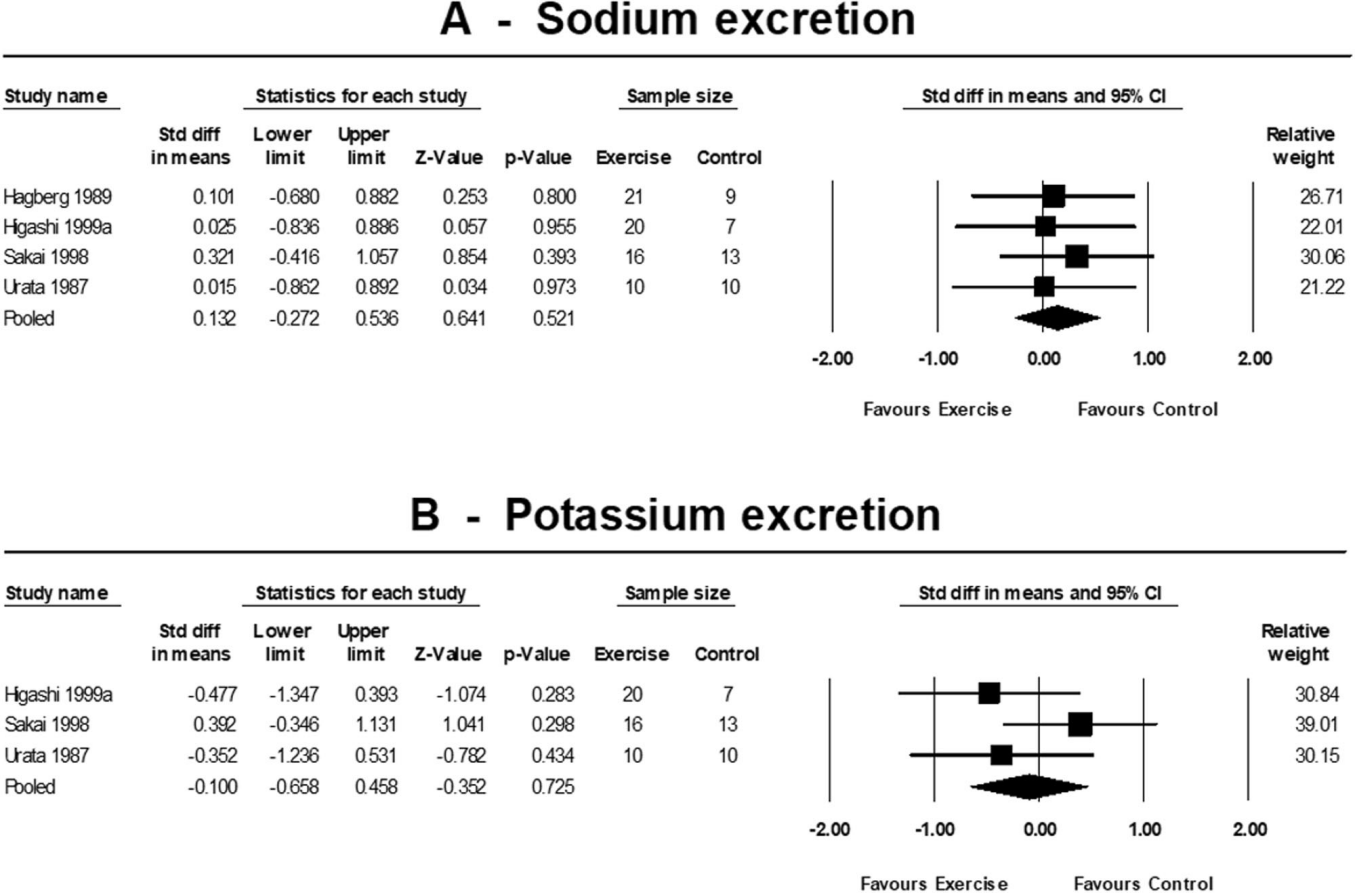
Standardised change in means of electrolytes. Forest plots showing the effects of exercise training on 24-h sodium [mmol/d] (**A**) and potassium [mmol/d] (**B**) excretion compared with control. A *p*-value < 0.05 represents a significant pooled standardised difference in means of overall effect. Horizontal lines across each present 95% CI for each study. The diamond represents the 95% CI for pooled estimates of effect of standardised mean difference.

The electrolytes assessed included the 24-h urinary sodium and potassium excretion, the recommended gold standard method of measuring an individual’s associations between sodium, potassium and BP [42]. There was no change in sodium excretion (4 trials); SMD 0.13 (95% CI −0.27 to 0.54, *p* = 0.521; *I*^2^ = 0%, *p* = 0.943) and potassium excretion (3 trials); SMD −0.10 (95% CI −0.66 to 0.46, *p* = 0.725; *I*^2^ = 26.9%, *p* = 0.255) (Fig. 4).

### Effect of exercise training on resting blood pressure and heart rate

Overall, exercise training had a significant effect on resting BP but not HR. Systolic BP reduced by a mean difference (MD) −6.2 mmHg (95% CI −9.9 to −2.6, *p* = 0.001; *I*^2^ = 85.4, *p* < 0.001) and diastolic BP by MD −4.5 mmHg (95% CI −6.9 to −2.1, *p* < 0.001; *I*^2^ = 92.5, *p* < 0.001) but resting HR MD −3.0 bpm (95% CI −6.0 to 0.4, *p* = 0.053; *I*^2^ = 91.3, *p* < 0.001) was unchanged (see Supplementary Fig. S1). For studies that reported on blood pressure measures alongside markers of RAAS, the decrease in SBP and DBP was significant for all parameters with the exception of aldosterone and epinephrine. Table 2 summarises the results of exercise training effect on resting BP for all the included studies and for studies reporting on BP alongside each of the outcome parameters (see Supplementary Figs. S2 and S3).

**Table 2:** Mean net changes in resting blood pressure for studies reporting BP alongside RAAS parameters.

Studies reporting BP data alongside RAAS parameters	*N*	Systolic blood pressure (mmHg)			Diastolic blood pressure (mmHg)		
		Mean net change (95% CI; *p-*value)	Heterogeneity		Mean net change (95% CI; *p*-value)	Heterogeneity	
			*I*^2^ (%)	*p-*value		*I*^2^ (%)	*p-*value
Overall	14	**−6.24 (−9.88 to −2.61; 0.001)**	85.38	0.000	**−4.48 (−6.90 to −2.06; 0.000)**	92.51	0.000
Angiotensin-II	7	**−5.95 (−10.31 to −1.58; 0.008)**	85.34	0.000	**−5.0 (−9.42 to −0.59; 0.026)**	94.28	0.000
Aldosterone	5	−9.14 (−18.2 to −0.09; 0.048)	80.92	0.000	−3.46 (−7.74 to 0.81; 0.112)	77.34	0.001
Plasma renin activity	6	**−9.40 (−15.84 to −2.96; 0.004)**	80.22	0.000	**−4.96(−8.76 to −1.15; 0.011)**	91.59	0.000
Norepinephrine	7	**−8.16 (−13.99 to −2.32; 0.006)**	76.82	0.000	**−4.25 (−7.71 to −0.79; 0.016)**	90.18	0.000
Epinephrine	5	−9.23 (−18.69 to 0.23; 0.056)	82.97	0.000	−3.74 (−8.02 to 0.54; 0.087)	93.21	0.000
Sodium excretion	4	**−8.17 (−12.24 to −4.10; 0.000)**	0	0.494	**−4.99 (−7.96 to −2.03; 0.001)**	0	0.771
Potassium excretion	3	**−8.59 (−13.16 to −4.03; 0.000)**	8.46	0.335	**−5.03 (−8.35 to −1.71; 0.003)**	0	0.570

Significant net changes are indicated in bold.

### Sensitivity analyses and sub-analyses

‘One study removed’ sensitivity analysis for RAAS parameters, systolic and diastolic BP and heart rate did not indicate that any study overly impacted results (Supplementary Fig. S4). Sub-analyses for the RAAS outcome measures angiotensin-II, aldosterone, norepinephrine and also systolic and diastolic BP when analysed by health status showed reductions in unhealthy participants. This was particularly pronounced in participants with hypertension and those taking medications. Sub-analyses of exercise interventions also showed that aerobic exercise training was more effective in reducing the RAAS outcomes, SBP and DBP than other forms of training (Supplementary Tables S3–S7 and Figs. S5–S9).

### Heterogeneity and publication bias

Statistical heterogeneity across most outcomes was moderate to high. Funnel plots were produced for all parameters (Supplementary Fig. S10). When assessed using Egger’s regression test, visual inspection of the funnel plots showed little evidence of publication bias.

### Study quality

Study quality was assessed using the TESTEX scale. Of a maximum 15-point scale the median score was 9 (range 8–12) with higher scores indicating better quality (Supplementary Table S2).

## Discussion

The main findings of this meta-analysis demonstrated that angiotensin-II and aldosterone were reduced with exercise training; however, plasma renin activity was unchanged. Plasma norepinephrine decreased but epinephrine was unchanged. There was no change in both 24-h urinary sodium and potassium excretion following exercise training. Collectively, the 14 studies that reported both BP and RAAS markers showed a reduction in overall systolic and diastolic BP, but not HR, after exercise training.

### Effect of exercise training on the renin–angiotensin–aldosterone system

In the present study, our pooled data analyses showed exercise training reduced plasma angiotensin-II and aldosterone. This contrasts with the findings reported in the previous meta-analysis [18], and may be due to Goessler et al. including only healthy populations, as angiotensin-II and aldosterone are likely to be elevated in people with heart failure and to a lesser extent in people with hypertension (these populations were subjects of our sub-analyses). The previous study [18] could also be underpowered considering the number of included studies (*n* = 3 each) for both analyses of angiotensin-II and aldosterone. In contrast, our meta-analysis depicted unchanged plasma renin activity with exercise training while the previous study found reductions. It could be argued that possible renin-independent mechanisms are involved in angiotensin-II formation, with suggested mechanisms such as prorenin activating the prorenin receptor to generate angiotensin-II independent of renin activation [43] and tissue-derived renin to generate angiotensin-II in the circulation [44, 45]. Arnold et al. [46] tested this hypothesis administering the angiotensin-II type 1 (AT_1_) receptor blocker losartan (50 mg) to older adults with autonomic failure hypertension. Unexpectedly, these authors found increased circulating angiotensin-II levels in people with hypertension and autonomic failure compared with matched healthy controls despite low plasma renin activity; they suggested the formation of additional angiotensin-II was independent of plasma renin activity. In addition, plasma renin activity which is actually a measure of the rate of generation of angiotensin-II in circulation, is usually also affected by environmental factors including lifestyle behaviours [47–50]. Our pooled data analysis showed no overall change in plasma renin activity, although, some individual studies have reported a reduction in plasma renin activity [22, 32, 51, 52], while others reported no change after exercise training [15, 28, 34–37, 53]. It is of note that the exercise-related reductions in BP have not been fully elucidated as a combination of different mechanisms are involved which differ in individuals/populations as well as the different exercise training protocols [12, 54, 55]. For example, long-term exercise training induces possible changes in vascular responsiveness as the role of endothelium-driven factors is thought to play important roles in the local regulation of vascular resistance [55]. Nitric oxide, a potent vasodilator, increased [32, 56] and endothelium-1, a potent vasoconstrictor, reduced [32, 57, 58] following exercise training. Additionally, it could be suggested that the timing of data collection in exercise studies, especially in individuals with high BP, may influence change in some cardiovascular variables such as BP and plasma renin activity. It is important to show BP and hormonal changes may be unrelated to humoral factors and plasma volume changes [15]. In patients with essential hypertension, Zhang et al. [59] found decreased BP, plasma renin activity and aldosterone after 1–4 weeks of low-to-moderate-intensity training, which remained stable from 4–10 weeks. These authors reported variations in depressor response in responders versus non-responders to exercise training, as determined by baseline mean BP and humoral factors, but also the involvement of genetics, in their multivariate analysis of the prognostic determinants of the depressor response to exercise therapy.

More importantly, the sub-analyses for angiotensin-II and aldosterone revealed exercise training produced reductions which seemed more effective in participants with hypertension than their healthy counterparts, of which aerobic exercise training was the most effective compared to the other modes of exercise training. Likewise, the sub-analyses of systolic and diastolic BP showed similar exercise training effects. Moreover, there were reductions in systolic and diastolic BP in studies also reporting individual plasma renin activity, and angiotensin-II, but not in studies measuring aldosterone. The non-significant net change in BP for studies also reporting aldosterone may be because 5 [31–33, 37, 38] out of the 8 studies included in the aldosterone meta-analysis did not report a reduction in aldosterone.

### Effect of exercise training on sympathetic nervous system activity and electrolytes

Assessing the effect of exercise training on SNS activity, our meta-analyses showed a reduction in norepinephrine but not epinephrine. The non-significant reduction in epinephrine may be due to insufficient statistical power due to the small number of studies that assessed epinephrine (5 studies, *n* = 136 exercise and 93 control group). Regardless of the latter, it could be speculated that improved baroreflex control may have occurred, which would lead to a reduction in sympathetic activity following exercise training [60]. Regular aerobic exercise also enhances endothelial function with ageing in men by reducing oxidative stress and preserving NO bioavailability [61]. Muscle sympathetic nerve activity is favourably altered in people with impaired metaboreflex, mostly resulting from long-term interventions (>16 weeks) including aerobic exercise of moderate to high intensity, performed in isolation or within multimodal training [62].

The reduction in norepinephrine level could be explained by the positive modulation of the autonomic balance by the exercise training effect [35, 63]. Moreover, in humans the plasma epinephrine response to exercise is small compared with that of norepinephrine [64]. Levels of plasma epinephrine increase only when exercise training results in hypoglycaemia at very high intensities [64, 65]. At that point, significant post-training effects may be observed. As such, almost all of the exercise programs adopted in the included studies were of low-to-moderate/vigorous relative intensities (that is, <85% HR_max_) which were unlikely to effect a large change in epinephrine levels. Regardless, both relative (subjective measure of exercise intensity specific to an individual’s level of fitness which is based on maximum exercise capacity) and absolute (objective measure) exercise intensity plays a major role in determining the SNS response to exercise training. Thus, the variations in terms of exercise intensity in measuring and comparing activity patterns as well as tracking them over time can introduce some elements of confusion and inconsistency [66]. Assessing the effect of exercise intensity was beyond the scope of this study. Despite the detection of a highly significant reduction in norepinephrine concentration, our meta-analysis of included studies showed no change in resting HR. It could be speculated that either the relative intensity or the duration of the exercise protocols adopted by the majority of the included studies were insufficient to elicit a decreased HR. Considering almost all the participants were >50 years old, exercise training-induced adaptations are potentially affected by aging due to structural and functional changes including a reduction in neuroendocrine function resulting in a decreased responsiveness to homoeostasis disruption [67]. Thus, the aging cardiovascular system may be protected by the benefits of chronic exercise-induced adaptation. A longer training duration (that is from 30 weeks or more) would probably be necessary for older individuals to show a reduction in resting HR [68, 69]. Individual studies [22, 35] with longer training durations (>30 weeks) showed decreases in norepinephrine and a reduction in HR. In hypertensive participants, Hagberg et al. [22] showed reductions in resting HR (8 beats/min; *p* < 0.01) and norepinephrine (57 pg/mL; *p* < 0.05) following 9 months of training. Likewise, in chronic heart failure patients, Passino et al. [35] showed reductions in resting HR (from 6 beats/min; *p* = 0.002) and norepinephrine (from 160 ng/L; *p* < 0.001) after 9 months of training. Contrary to 10 weeks of training in young healthy adults (mean age 28 years), Greiwe et al. [64] found increases in both epinephrine and norepinephrine at increased exercise intensities with no change in HR comparing before and after training.

The two main electrolytes assessed in the present study were urinary sodium and potassium excretion. The analyses of both 24-h urinary sodium and potassium excretion in exercise vs control groups was unchanged. The reason for this cannot be explained even though our meta-analysis demonstrated a reduction in aldosterone, although there was decreased statistical power due to low number of studies included (that is, for sodium and potassium excretion: 4 and 3 studies, respectively) in the analyses. However, the slight increase in sodium excretion versus the reduction in potassium excretion might suggest improved sodium/potassium exchange following the cumulative effects of chronic exercise training which may have lowered aldosterone level. Aldosterone plays a major role in the regulation sodium reabsorption and potassium secretion [70–72]. During prolonged exercise, the body loses water and electrolytes as sweat. Sweating is influenced by several factors that increase in proportion to the rate of workload (including intensity, volume, duration per session or type of exercise) and the environmental temperature, humidity [73, 74] and there is individual variability [75]. Studies have shown that intracellular and plasma (or serum) sodium are higher in hypertensives than their normotensives counterparts [76–80]. The electrolyte responses to exercise training in hypertensives and normotensives may be different due to genetic abnormalities in body fluid and electrolyte homoeostasis [37, 81].

### Limitations

This study had limitations including the small sample sizes in the included studies for some measurements which may have been underpowered. In addition, we could not establish a relationship between exercise-induced changes in BP and changes in outcome measures of interest. We acknowledge that only included studies that measured markers of RAAS as well as BP and HR were included in the review, indicating that the impact of exercise training on systemic hemodynamics is likely not reflected by this study. This analysis was also limited by the varied health and medication status of participants. Another possible limitation of included studies is that none of the studies controlled participants’ dietary intake (e.g., sodium).

## Conclusion

Our meta-analysis showed that exercise training led to a reduction in some RAAS parameters (angiotensin-II and aldosterone), SNS activity (norepinephrine) and overall BP. Exercise training may induce a reduction in some aspects of RAAS activity and this might play a vital role in the post-training BP response despite the unobserved change in plasma renin activity. Given the limited study data available for the assessment of changes in the parameters of RAAS, SNS activity and electrolytes, future research is warranted to evaluate the role of RAAS, SNS activity and electrolytes in BP response following exercise training and possible relationship between BP changes.

### Supplementary information

Supplementary Information

## Data Availability

All data generated and analysed are included in this article and the associated online resources.

## References

[1] Zhou B, Perel P, Mensah GA, Ezzati M (2021). Global epidemiology, health burden and effective interventions for elevated blood pressure and hypertension. Nat Rev Cardiol.

[2] Morrow JR, Krzewinski-Malone JA, Jackson AW, Bungum TJ, FitzGerald SJ (2004). American adults’ knowledge of exercise recommendations. Res Q Exerc Sport.

[3] Warburton DE, Nicol CW, Bredin SS (2006). Health benefits of physical activity: the evidence. CMAJ.

[4] Cornelissen VA, Buys R, Smart NA (2013). Endurance exercise beneficially affects ambulatory blood pressure: a systematic review and meta-analysis. J Hypertens.

[5] Cornelissen VA, Fagard RH (2005). Effects of endurance training on blood pressure, blood pressure-regulating mechanisms, and cardiovascular risk factors. Hypertension.

[6] Sherwood JJ, Inouye C, Webb SL, Zhou A, Anderson EA, Spink NS (2019). Relationship between physical and cognitive performance in community dwelling, ethnically diverse older adults: a cross-sectional study. PeerJ.

[7] Monteiro MDF, Sobral Filho DC (2004). Physical exercise and blood pressure control. Rev Brasil Med Esport.

[8] Fallo F (1993). Renin-angiotensin-aldosterone system and physical exercise. J Sports Med Phys Fit.

[9] Bernal J, Pitta SR, Thatai D (2006). Role of the renin-angiotensin-aldosterone system in diastolic heart failure: potential for pharmacologic intervention. Am J Cardiovasc Drugs.

[10] Klabunde RE. Renin-angiotensin-aldosterone system. In: Cardiovascular physiology concepts. 3rd ed. Wolters Kluwer; Philadelphia: 2021. https://www.cvphysiology.com.

[11] Gomes-Santos IL, Fernandes T, Couto GK, Ferreira-Filho JCA, Salemi VMC, Fernandes FB (2014). Effects of exercise training on circulating and skeletal muscle renin-angiotensin system in chronic heart failure rats. PLoS ONE.

[12] MacDonald JR (2002). Potential causes, mechanisms, and implications of post exercise hypotension. J Hum Hypertens.

[13] Magalhães DM, Nunes-Silva A, Rocha GC, Vaz LN, de Faria MHS, Vieira ELM (2020). Two protocols of aerobic exercise modulate the counter-regulatory axis of the renin-angiotensin system. Heliyon.

[14] Ciocoiu M, Bararu-Bojan I, Vladeanu M, Badescu C. The renin-angiotensin-aldosterone system: genomics, proteomics and therapeutic implications. In: Kibel A, editor. Selected Chapters from the Renin-Angiotensin System. IntechOpen; Rijeka: 2020. 10.5772/intechopen.77666.

[15] Collier S, Sandberg K, Moody A, Frechette V, Curry CD, Ji H (2014). Reduction of plasma aldosterone and arterial stiffness in obese pre- and stage1 hypertensive subjects after aerobic exercise. J Hum Hypertens.

[16] Patel S, Rauf A, Khan H, Abu-Izneid T (2017). Renin-angiotensin-aldosterone (RAAS): the ubiquitous system for homeostasis and pathologies. Biomed Pharmacother.

[17] Fountain JH, Lappin SL. Physiology, renin angiotensin system. In: StatPearls [Internet]. Treasure Island, FL: StatPearls Publishing. Available from: https://www.ncbi.nlm.nih.gov/books/NBK470410/. 29261862

[18] Goessler K, Polito M, Cornelissen VA (2016). Effect of exercise training on the renin–angiotensin–aldosterone system in healthy individuals: a systematic review and meta-analysis. Hypertens Res.

[19] Baffour-Awuah B, Man M, Goessler KF, Cornelissen V, Pearson M, Dieberg G, et al. Effect of exercise training on the renin–angiotensin–aldosterone system: a systematic review and meta-analysis. PROSPERO. 2021. https://www.crd.york.ac.uk/prospero/display_record.php?ID=CRD42021255225. 10.1038/s41371-023-00872-4 PMC10844078 38017087

[20] Rohatgi A. WebPlotDigitizer Version 4.5 [Computer software] 2021. Available from: https://automeris.io/WebPlotDigitizer.

[21] Cortez-Cooper MY, Anton MM, Devan AE, Neidre DB, Cook JN, Tanaka H (2008). The effects of strength training on central arterial compliance in middle-aged and older adults. Eur J Cardiovasc Prev Rehabil.

[22] Hagberg JM, Montain SJ, Martin WH, Ehsani AA (1989). Effect of exercise training in 60- to 69-year-old persons with essential hypertension. Am J Cardiol.

[23] Higgins JP, Thompson SG, Deeks JJ, Altman DG (2003). Measuring inconsistency in meta-analyses. Br Med J.

[24] Egger M, Smith GD, Schneider M, Minder C (1997). Bias in meta-analysis detected by a simple, graphical test. Br Med J.

[25] Smart NA, Waldron M, Ismail H, Giallauria F, Vigorito C, Cornelissen V (2015). Validation of a new tool for the assessment of study quality and reporting in exercise training studies: TESTEX. Int J Evid Based Health.

[26] Anton MM, Cortez-Cooper MY, DeVan AE, Neidre DB, Cook JN, Tanaka H (2006). Resistance training increases basal limb blood flow and vascular conductance in aging humans. J Appl Physiol.

[27] Azadpour N, Tartibian B, Koşar ŞN (2017). Effects of aerobic exercise training on ACE and ADRB2 gene expression, plasma angiotensin II level, and flow-mediated dilation: a study on obese postmenopausal women with prehypertension. Menopause.

[28] Bilińska M, Kosydar-Piechna M, Mikulski T, Piotrowicz E, Gąsiorowska A, Piotrowski W (2013). Influence of aerobic training on neurohormonal and hemodynamic responses to head-up tilt test and on autonomic nervous activity at rest and after exercise in patients after bypass surgery. Cardiol J.

[29] Braith RW, Welsch MA, Feigenbaum MS, Kluess HA, Pepine CJ (1999). Neuroendocrine activation in heart failure is modified by endurance exercise training. J Am Coll Cardiol.

[30] Brubaker PH, Moore JB, Stewart KP, Wesley DJ, Kitzman DW (2009). Endurance exercise training in older patients with heart failure: results from a randomized, controlled, single-blind trial. J Am Geriatr Soc.

[31] Carroll JF, Convertino VA, Wood CE, Graves JE, Lowenthal DT, Pollock ML (1995). Effect of training on blood volume and plasma hormone concentrations in the elderly. Med Sci Sports Exerc.

[32] Cruz LG, Bocchi EA, Grassi G, Guimaraes GV (2017). Neurohumoral and endothelial responses to heated water-based exercise in resistant hypertensive patients. Circ J.

[33] Higashi Y, Sasaki S, Kurisu S, Yoshimizu A, Sasaki N, Matsuura H (1999). Regular aerobic exercise augments endothelium-dependent vascular relaxation in normotensive as well as hypertensive subjects. Circulation.

[34] Higashi Y, Sasaki S, Sasaki N, Nakagawa K, Ueda T, Yoshimizu A (1999). Daily aerobic exercise improves reactive hyperemia in patients with essential hypertension. Hypertension.

[35] Passino C, Severino S, Poletti R, Piepoli MF, Mammini C, Clerico A (2006). Aerobic training decreases B-type natriuretic peptide expression and adrenergic activation in patients with heart failure. J Am Coll Cardiol.

[36] Sakai T, Ideishi M, Miura S, Maeda H, Tashiro E, Koga M (1998). Mild exercise activates renal dopamine system in mild hypertensives. J Hum Hypertens.

[37] Urata H, Tanabe Y, Kiyonaga A, Ikeda M, Tanaka H, Shindo M (1987). Antihypertensive and volume-depleting effects of mild exercise on essential hypertension. Hypertension.

[38] Waib PH, Gonçalves MI, Barrile SR (2011). Improvements in insulin sensitivity and muscle blood flow in aerobic-trained overweight-obese hypertensive patients are not associated with ambulatory blood pressure. J Clin Hypertens.

[39] Yoshizawa M, Maeda S, Miyaki A, Misono M, Choi Y, Shimojo N (2009). Additive beneficial effects of lactotripeptides and aerobic exercise on arterial compliance in postmenopausal women. Am J Physiol Heart Circ Physiol.

[40] Corrêa HL, Neves RVP, Deus LA, Maia BCH, Maya AT, Tzanno-Martins C (2021). Low-load resistance training with blood flow restriction prevent renal function decline: the role of the redox balance, angiotensin 1–7 and vasopressin*,**. Physiol Behav.

[41] Lin B, Jin Q, Liu C, Zhao W, Ji R (2022). Effect and mechanism of tai chi on blood pressure of patients with essential hypertension: a randomized controlled study. J Sports Med Phys Fit.

[42] Morrissey E, Giltinan M, Kehoe L, Nugent AP, McNulty BA, Flynn A (2020). Sodium and potassium intakes and their ratio in adults (18–90 y): findings from the Irish national adult nutrition survey. Nutrients.

[43] Nguyen G, Delarue F, Burcklé C, Bouzhir L, Giller T, Sraer JD (2002). Pivotal role of the renin/prorenin receptor in angiotensin II production and cellular responses to renin. J Clin Invest.

[44] Bader M (2010). Tissue renin-angiotensin-aldosterone systems: targets for pharmacological therapy. Annu Rev Pharm Toxicol.

[45] Nehme A, Zouein FA, Zayeri ZD, Zibara K (2019). An update on the tissue renin angiotensin system and its role in physiology and pathology. J Cardiovasc Dev Dis.

[46] Arnold AC, Okamoto LE, Gamboa A, Shibao C, Raj SR, Robertson D (2013). Angiotensin II, independent of plasma renin activity, contributes to the hypertension of autonomic failure. Hypertension.

[47] Cartledge S, Lawson N (2000). Aldosterone and renin measurements. Ann Clin Biochem.

[48] Pizoń T, Rajzer M, Wojciechowska W, Drożdż T, Drożdż D, Rojek M (2021). Plasma renin activity, serum aldosterone concentration and selected organ damage indices in essential arterial hypertension. Arch Med Sci.

[49] Patlar S, ÜNsal S (2019). RAA system and exercise relationship. Turk J Sport Exerc.

[50] Allikmets K, Parik T, Viigimaa M (1999). The renin-angiotensin system in essential hypertension: associations with cardiovascular risk. Blood Press.

[51] Geyssant A, Geelen G, Denis C, Allevard AM, Vincent M, Jarsaillon E (1981). Plasma vasopressin, renin activity, and aldosterone: effect of exercise and training. Eur J Appl Physiol Occup Physiol.

[52] Jennings G, Nelson L, Nestel P, Esler M, Korner P, Burton D (1986). The effects of changes in physical activity on major cardiovascular risk factors, hemodynamics, sympathetic function, and glucose utilization in man: a controlled study of four levels of activity. Circulation.

[53] Hespel P, Lijnen P, Van Hoof R, Fagard R, Goossens W, Lissens W (1988). Effects of physical endurance training on the plasma renin-angiotensin-aldosterone system in normal man. J Endocrinol.

[54] Grotle AK, Macefield VG, Farquhar WB, O’Leary DS, Stone AJ (2020). Recent advances in exercise pressor reflex function in health and disease. Auton Neurosci.

[55] Hamer M (2006). The anti-hypertensive effects of exercise. Sports Med.

[56] Lewis TV, Dart AM, Chin-Dusting JPF, Kingwell BA (1999). Exercise training increases basal nitric oxide production from the forearm in hypercholesterolemic patients. Arterioscler Thromb Vasc Biol.

[57] Guilder GPV, Westby CM, Greiner JJ, Stauffer BL, DeSouza CA (2007). Endothelin-1 vasoconstrictor tone increases with age in healthy men but can be reduced by regular aerobic exercise. Hypertension.

[58] Maeda S, Tanabe T, Miyauchi T, Otsuki T, Sugawara J, Iemitsu M (2003). Aerobic exercise training reduces plasma endothelin-1 concentration in older women. J Appl Physiol.

[59] Zhang B, Sakai T, Noda K, Kiyonaga A, Tanaka H, Shindo M (2003). Multivariate analysis of the prognostic determinants of the depressor response to exercise therapy in patients with essential hypertension. Circ J.

[60] Cruz RSDO, de Aguiar RA, Turnes T, Salvador AF, Caputo F (2016). Effects of ischemic preconditioning on short-duration cycling performance. Appl Physiol Nutr Metab.

[61] Seals DR, Nagy EE, Moreau KL (2019). Aerobic exercise training and vascular function with ageing in healthy men and women. J Physiol.

[62] Gama G, Farinatti P, Rangel M, Mira PAC, Laterza MC, Crisafulli A (2021). Muscle metaboreflex adaptations to exercise training in health and disease. Eur J Appl Physiol.

[63] Coats AJ, Adamopoulos S, Radaelli A, McCance A, Meyer TE, Bernardi L (1992). Controlled trial of physical training in chronic heart failure. Exercise performance, hemodynamics, ventilation, and autonomic function. Circulation.

[64] Greiwe JS, Hickner RC, Shah SD, Cryer PE, Holloszy JO (1999). Norepinephrine response to exercise at the same relative intensity before and after endurance exercise training. J Appl Physiol.

[65] Cryer PE (1980). Physiology and pathophysiology of the human sympathoadrenal neuroendocrine system. N Engl J Med.

[66] Norton K, Norton L, Sadgrove D (2010). Position statement on physical activity and exercise intensity terminology. J Sci Med Sport.

[67] Silverman HG, Mazzeo RS (1996). Hormonal responses to maximal and submaximal exercise in trained and untrained men of various ages. J Gerontol A Biol Sci Med Sci.

[68] Huang G, Shi X, Davis-Brezette JA, Osness WH (2005). Resting heart rate changes after endurance training in older adults: a meta-analysis. Med Sci Sports Exerc.

[69] Kiyonaga A, Arakawa K, Tanaka H, Shindo M (1985). Blood pressure and hormonal responses to aerobic exercise. Hypertension.

[70] Prasertsri P, Singsanan S, Chonanant C, Boonla O, Trongtosak P (2019). Effects of arm swing exercise training on cardiac autonomic modulation, cardiovascular risk factors, and electrolytes in persons aged 60-80 years with prehypertension: A randomized controlled trial. J Exerc Sci Fit.

[71] Scott JH, Menouar MA, Dunn RJ. Physiology, aldosterone. In: StatPearls [Internet]. Treasure Island, FL: StatPearls Publishing. Available from: https://www.ncbi.nlm.nih.gov/books/NBK470339/#_NBK470339_pubdet_. 29261963

[72] Hu G, Xu X, Liang X, Yang X, Zhang J, Simayi Z (2013). Associations of plasma atrial natriuretic peptide and electrolyte levels with essential hypertension. Exp Ther Med.

[73] Maughan RJ (1991). Fluid and electrolyte loss and replacement in exercise. J Sports Sci.

[74] Baker LB, De Chavez PJD, Ungaro CT, Sopena BC, Nuccio RP, Reimel AJ (2019). Exercise intensity effects on total sweat electrolyte losses and regional vs. whole-body sweat [Na(+)], [Cl(-)], and [K(+)]. Eur J Appl Physiol.

[75] Lara B, Gallo-Salazar C, Puente C, Areces F, Salinero JJ, Del Coso J (2016). Interindividual variability in sweat electrolyte concentration in marathoners. J Int Soc Sports Nutr.

[76] Geleijnse JM, Kok FJ, Grobbee DE (2003). Blood pressure response to changes in sodium and potassium intake: a metaregression analysis of randomised trials. J Hum Hypertens.

[77] Kogure M, Nakaya N, Hirata T, Tsuchiya N, Nakamura T, Narita A (2021). Sodium/potassium ratio change was associated with blood pressure change: possibility of population approach for sodium/potassium ratio reduction in health checkup. Hypertens Res.

[78] Ndanuko RN, Ibrahim R, Hapsari RA, Neale EP, Raubenheimer D, Charlton KE (2021). Association between the urinary sodium to potassium ratio and blood pressure in adults: a systematic review and meta-analysis. Adv Nutr.

[79] Nwachukwu D, Aneke E, Nwachukwu N, Obika L, Nwagha U, Eze A (2015). Effect of Hibiscus sabdariffa on blood pressure and electrolyte profile of mild to moderate hypertensive Nigerians: a comparative study with hydrochlorothiazide. Niger J Clin Pr.

[80] Whelton PK (2014). Sodium, potassium, blood pressure, and cardiovascular disease in humans. Curr Hypertens Rep.

[81] Beretta-Piccoli C, Davies DL, Boddy K, Brown JJ, Cumming AM, East WB (1981). Relation of arterial pressure with exchangeable and total body sodium and with plasma exchangeable and total body potassium in essential hypertension. Clin Sci.

