# Supplementary material for: Effect of exercise training on the renin–angiotensin–aldosterone system: a meta–analysis
Source: J Hum Hypertens. 2023 Nov 28;38(2):89–101. doi: 10.1038/s41371-023-00872-4 (PMC10844078; doi:10.1038/s41371-023-00872-4)
Supplement: Supplementary file 1 — Supplementary Information [file 41371_2023_872_MOESM1_ESM.docx]

Journal of Human Hypertension

**Effect of exercise training on the renin–angiotensin–aldosterone system: a systematic review and meta-analysis**

Biggie Baffour-Awuah, Melody Man, Karla F Goessler, Véronique A Cornelissen, Gudrun Dieberg, Neil A Smart, and Melissa J Pearson

**SUPPLEMENTARY INFORMATION**

**Supplementary Table S1: Search strategy (PubMed)**

| Items | Query |
| --- | --- |
| #5 | Search: ((((((((aerobic exercise[Title/Abstract]) OR (resistance exercise[Title/Abstract])) OR (exercise[Title/Abstract])) OR (training[Title/Abstract])) OR (physical activity[Title/Abstract])) OR (isometric exercise[Title/Abstract])) OR (endurance training[Title/Abstract])) OR (strength training[Title/Abstract])) AND (((((renin angiotensin aldosterone system[Title/Abstract]) OR (renin angiotensin system[Title/Abstract])) OR (renin[Title/Abstract])) OR (angiotensin[Title/Abstract])) OR (aldosterone[Title/Abstract])) Filters: Controlled Clinical Trial, Randomized Controlled Trial, Humans |
| #4 | Search: ((((((((aerobic exercise[Title/Abstract]) OR (resistance exercise[Title/Abstract])) OR (exercise[Title/Abstract])) OR (training[Title/Abstract])) OR (physical activity[Title/Abstract])) OR (isometric exercise[Title/Abstract])) OR (endurance training[Title/Abstract])) OR (strength training[Title/Abstract])) AND (((((renin angiotensin aldosterone system[Title/Abstract]) OR (renin angiotensin system[Title/Abstract])) OR (renin[Title/Abstract])) OR (angiotensin[Title/Abstract])) OR (aldosterone[Title/Abstract])) Filters: Controlled Clinical Trial, Randomized Controlled Trial |
| #3 | Search: ((((((((aerobic exercise[Title/Abstract]) OR (resistance exercise[Title/Abstract])) OR (exercise[Title/Abstract])) OR (training[Title/Abstract])) OR (physical activity[Title/Abstract])) OR (isometric exercise[Title/Abstract])) OR (endurance training[Title/Abstract])) OR (strength training[Title/Abstract])) AND (((((renin angiotensin aldosterone system[Title/Abstract]) OR (renin angiotensin system[Title/Abstract])) OR (renin[Title/Abstract])) OR (angiotensin[Title/Abstract])) OR (aldosterone[Title/Abstract])) |
| #2 | Search: ((((renin angiotensin aldosterone system[Title/Abstract]) OR (renin angiotensin system[Title/Abstract])) OR (renin[Title/Abstract])) OR (angiotensin[Title/Abstract])) OR (aldosterone[Title/Abstract]) |
| #1 | Search: (((((((aerobic exercise[Title/Abstract]) OR (resistance exercise[Title/Abstract])) OR (exercise[Title/Abstract])) OR (training[Title/Abstract])) OR (physical activity[Title/Abstract])) OR (isometric exercise[Title/Abstract])) OR (endurance training[Title/Abstract])) OR (strength training[Title/Abstract]) |

This search strategy was repeated for the additional data bases used; Web of Science and Cochrane Library of Controlled Trials.

**Supplementary Table S2: TESTEX scale assessment of included studies**

| **Study** | **Eligibility criteria specified** | **Randomization specified** | **Allocation concealment** | **Groups similar at baseline** | **Blinding of assessor** | **Outcome measures assessed >85% of participants^#^** | **Intention to treat analysis** | **Between group statistical comparisons reported^*^** | **Point measures & measures of variability reported** | **Activity monitoring in control group** | **Relative exercise intensity review** | **Exercise volume & energy expenditure** | **Overall TESTEX** |
| --- | --- | --- | --- | --- | --- | --- | --- | --- | --- | --- | --- | --- | --- |
| Anton et al. 2006 (1) | 1 | 0 | 0 | 1 | 0 | 2 | 1 | 2 | 1 | 1 | 1 | 0 | 10 |
| Azadpour et al. 2017 (2) | 1 | 0 | 0 | 1 | 0 | 2 | 1 | 2 | 1 | 0 | 1 | 0 | 9 |
| Bilińska et al. 2013 (3) | 1 | 0 | 0 | 1 | 0 | 2 | 1 | 2 | 1 | 0 | 1 | 0 | 9 |
| Braith et al. 1999 (4) | 1 | 1 | 0 | 1 | 0 | 2 | 1 | 2 | 1 | 1 | 1 | 0 | 11 |
| Brubaker et al. 2009 (5) | 1 | 0 | 0 | 1 | 1 | 3 | 0 | 2 | 1 | 0 | 1 | 0 | 10 |
| Carroll et al. 1995 (6) | 1 | 0 | 0 | 1 | 0 | 2 | 0 | 2 | 1 | 0 | 1 | 0 | 8 |
| Correa et al. 2021 (7) | 1 | 0 | 0 | 1 | 0 | 2 | 1 | 2 | 1 | 0 | 1 | 0 | 9 |
| Cortez-Cooper et al. 2008 (8) | 1 | 0 | 0 | 1 | 0 | 2 | 0 | 2 | 1 | 1 | 1 | 0 | 9 |
| Cruz et al. 2017 (9) | 1 | 1 | 1 | 1 | 0 | 3 | 1 | 2 | 1 | 0 | 1 | 0 | 12 |
| Hagberg et al. 1989 (10) | 1 | 0 | 0 | 1 | 0 | 2 | 0 | 2 | 1 | 0 | 1 | 0 | 8 |
| Higashi et al. 1999a (11) | 1 | 0 | 0 | 1 | 0 | 2 | 1 | 2 | 1 | 0 | 0 | 0 | 8 |
| Higashi et al. 1999b (12) | 1 | 0 | 0 | 1 | 0 | 2 | 1 | 2 | 1 | 0 | 0 | 0 | 8 |
| Lin et al. 2022 (13) | 1 | 0 | 0 | 1 | 0 | 2 | 1 | 2 | 1 | 0 | 0 | 0 | 8 |
| Passino et al. 2006 (14) | 1 | 0 | 0 | 1 | 0 | 2 | 1 | 2 | 1 | 0 | 0 | 0 | 8 |
| Sakai et al. 1998 (15) | 1 | 0 | 0 | 1 | 0 | 2 | 1 | 2 | 1 | 0 | 1 | 0 | 9 |
| Urata et al. 1987 (16) | 1 | 0 | 0 | 1 | 0 | 2 | 1 | 2 | 1 | 0 | 1 | 0 | 9 |
| Waib et al. 2011 (17) | 1 | 1 | 0 | 0 | 0 | 2 | 1 | 2 | 1 | 1 | 1 | 0 | 10 |
| Yoshizawa et al. 2009 (18) | 1 | 0 | 0 | 1 | 0 | 2 | 1 | 2 | 1 | 0 | 1 | 0 | 9 |
| **Median Score** | | | | | | | | | | | | | **9** |

Total out of 15 Points

# Three points possible: 1 point – if adherence >85%, 1 point – if adverse events reported, 1 point – if exercise attendance is reported

* Two points possible: 1 point – if between-group statistical comparisons are reported for the primary outcome measure of interest, 1 point – if between-group statistical comparisons are reported for at least one secondary outcome measure

**Supplementary Table S3: Summary of sub-analyses of angiotensin-II** (also see Figure S5)

| **Moderator** | **No. of Studies** | **SMD** | **95% CI** | ***p*-value** | **Heterogeneity** | |
| --- | --- | --- | --- | --- | --- | --- |
|  |  |  |  |  | ***I*^2^** | **p-value** |
| **Health status** | | | | | | |
| Healthy | 4 | -1.15 | -2.60 – 0.30 | 0.120 | 90.88 | 0.000 |
| Unhealthy | 5 | **-0.59** | -0.92 – -0.26 | 0.000 | 28.66 | 0.230 |
| Heart failure | 2 | -0.75 | -1.97 – 0.47 | 0.227 | 77.17 | 0.036 |
| Hypertension | 3 | **-0.62** | -0.93 – -0.32 | 0.000 | 0 | 0.000 |
| Medication | | | | | | |
| No | 6 | **-0.83** | -1.64 – -0.01 | 0.047 | 85.47 | 0.000 |
| Yes | 3 | **-0.59** | -1.16 – -0.02 | 0.043 | 54.34 | 1.112 |
| **Exercise intervention** | | | | | | |
| Aerobic | 5 | **-1.22** | -2.43 – -0.01 | 0.047 | 88.68 | 0.000 |
| Resistance | 3 | -0.34 | -0.71 – 0.02 | 0.066 | 0 | 0.547 |
| Tai chi | 1 | **-0.75** | -1.16 – -0.35 | 0.000 | 0 | 1.000 |
| **Control activity** | | | | | | |
| Sedentary | 7 | **-0.92** | -1.57 – -0.26 | 0.006 | 83.28 | 0.000 |
| Stretching | 2 | -0.17 | -0.68 – 0.35 | 0.520 | 0 | 0.558 |

**Supplementary Table S4: Summary of sub-analyses of aldosterone** (also see Figure S6)

| **Moderator** | **No. of Studies** | **SMD** | **95% CI** | ***p*-value** | **Heterogeneity** | |
| --- | --- | --- | --- | --- | --- | --- |
|  |  |  |  |  | ***I*^2^** | **p-value** |
| **Health status** | | | | | | |
| Healthy | 1 | -0.08 | -0.84 – 0.68 | 0.842 | 0 | 1.000 |
| Unhealthy | 7 | **-0.41** | -0.72 – -0.11 | 0.009 | 35.98 | 0.154 |
| Heart failure | 3 | -0.62 | -1.32 – 0.09 | 0.088 | 72.14 | 0.028 |
| Hypertension | 4 | -0.32 | -0.65 – 0.01 | 0.059 | 0 | 0.556 |
| **Medication** | | | | | | |
| No | 4 | -0.22 | -0.56 – 0.13 | 0.222 | 0 | 0.604 |
| Yes | 4 | **-0.54** | -1.03 – -0.05 | 0.030 | 58.56 | 0.065 |
| **Exercise intervention** | | | | | | |
| Aerobic | 7 | **-0.37** | -0.69 – -0.04 | 0.026 | 37.4 | 0.143 |
| Water exercise | 1 | -0.49 | -1.11 – 0.14 | 0.126 | 0 | 1.000 |
| **Control activity** | | | | | | |
| Sedentary | 7 | **-0.44** | -0.76 – -0.12 | 0.008 | 32.25 | 0.182 |
| Callisthenics | 1 | -0.12 | -0.60 – 0.36 | 0.633 | 0 | 1.000 |

**Supplementary Table S5: Summary of sub-analyses of norepinephrine** (also see Figure S7)

| **Moderator** | **No. of Studies** | **MD** | **95% CI** | ***p*-value** | **Heterogeneity** | |
| --- | --- | --- | --- | --- | --- | --- |
|  |  |  |  |  | ***I*^2^** | **p-value** |
| **Health status** | | | | | | |
| Healthy | 1 | 0.16 | -0.63 – 0.95 | 0.688 | 0 | 1.000 |
| Unhealthy | 7 | **-0.94** | -1.25 – -0.64 | 0.000 | 35.12 | 0.160 |
| CAD | 1 | **-1.13** | -1.55 – -0.71 | 0.000 | 1 | 1.000 |
| Heart failure | 1 | **-0.73** | -1.17 – -0.29 | 0.001 | 1 | 1.000 |
| Hypertension | 5 | **-0.95** | -1.45 – -0.45 | 0.000 | 47.14 | 0.109 |
| **Medication** | | | | | | |
| No | 4 | -0.4 | -0.9 – 0.1 | 0.114 | 30.64 | 0.228 |
| Yes | 4 | **-1.09** | -1.43 – -0.74 | 0.000 | 35.18 | 0.201 |
| **Exercise intervention** | | | | | | |
| Aerobic | 7 | **-0.72** | -1.07 – -0.37 | 0.000 | 47.33 | 0.077 |
| Water exercise | 1 | **-1.6** | -2.3 – -0.9 | 0.000 | 0 | 1.000 |

**Supplementary Table S6: Summary of sub-analyses of systolic blood pressure** (also see Figure S8)

| **Moderator** | **No. of Studies** | **MD** | **95% CI** | ***p*-value** | **Heterogeneity** | |
| --- | --- | --- | --- | --- | --- | --- |
|  |  |  |  |  | ***I*^2^** | **p-value** |
| **Health status** | | | | | | |
| Healthy | 5 | -2.62 | -6.38 – 1.14 | 0.172 | 32.81 | 0.203 |
| Unhealthy | 9 | **-8.76** | -13.57 – -3.95 | 0.000 | 90.52 | 0.000 |
| CAD | 1 | -1.63 | -3.89 – 0.63 | 0.157 | 0 | 1.000 |
| Hypertension | 8 | **-9.76** | -14.17 – -5.35 | 0.000 | 81.21 | 0.000 |
| **Medication** | | | | | | |
| No | 10 | **-4.78** | -7.57 – -1.99 | 0.001 | 51.50 | 0.029 |
| Yes | 4 | **-11.27** | -20.7 – -1.85 | 0.019 | 95.92 | 0.000 |
| **Exercise intervention** | | | | | | |
| Aerobic | 9 | **-5.07** | -7.87 – -2.26 | 0.000 | 63.24 | 0.005 |
| Resistance | 3 | -3.72 | -16.74 – 9.3 | 0.575 | 91.58 | 0.000 |
| Tai chi | 1 | -6.0 | -13.42 – 1.42 | 0.113 | 0 | 1.000 |
| Water exercise | 1 | **-26.9** | -38.63 – -15.18 | 0.000 | 0 | 1.000 |
| **Control activity** | | | | | | |
| Sedentary | 11 | **-8.16** | -12.13 – -4.18 | 0.000 | 84.80 | 0.000 |
| Stretching/Calisthenics | 3 | 0.82 | -3.27 – 4.91 | 0.694 | 0 | 0.823 |

**Supplementary Table S7: Summary of sub-analyses of diastolic blood pressure** (also see Figure S9)

| **Moderator** | **No. of Studies** | **MD** | **95% CI** | ***p*-value** | **Heterogeneity** | |
| --- | --- | --- | --- | --- | --- | --- |
|  |  |  |  |  | ***I*^2^** | **p-value** |
| **Health status** | | | | | | |
| Healthy | 5 | **-2.39** | -3.33 – -1.45 | 0.000 | 0 | 0.914 |
| Unhealthy | 9 | **-5.79** | -9.50 – -2.09 | 0.002 | 94.99 | 0.000 |
| CAD | 1 | **-1.26** | -2.19 – -0.34 | 0.008 | 0 | 1.000 |
| Hypertension | 8 | **-6.56** | -9.98 – -3.14 | 0.000 | 88.46 | 0.000 |
| **Medication** | | | | | | |
| No | 10 | **-3.03** | -4.16 – -1.91 | 0.000 | 12.95 | 0.324 |
| Yes | 4 | -7.31 | -13.64 – -0.99 | 0.023 | 98.06 | 0.000 |
| **Exercise intervention** | | | | | | |
| Aerobic | 9 | **-2.49** | -3.66 – -1.33 | 0.000 | 44.39 | 0.072 |
| Resistance | 3 | -7.04 | -16.9 – 2.83 | 0.162 | 91.77 | 0.000 |
| Tai chi | 1 | **-8.0** | -14.53 – -1.47 | 0.016 | 0 | 1.000 |
| Water exercise | 1 | **-7.80** | -9.22 – -6.38 | 0.000 | 0 | 1.000 |
| **Control activity** | | | | | | |
| Sedentary | 11 | **-5.12** | -7.85 – -2.38 | 0.000 | 94.15 | 0.000 |
| Stretching/Calisthenics | 3 | -1.39 | -4.59 – 1.82 | 0.397 | 0 | 0.793 |

| 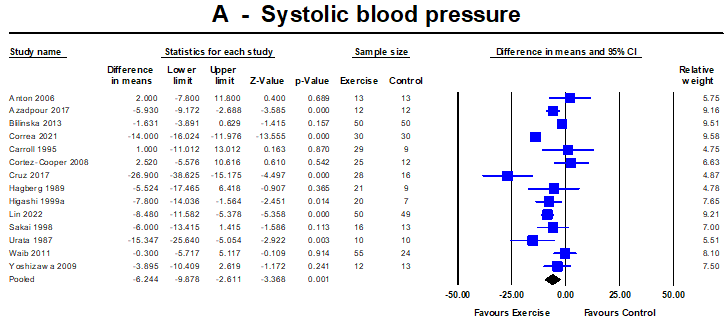  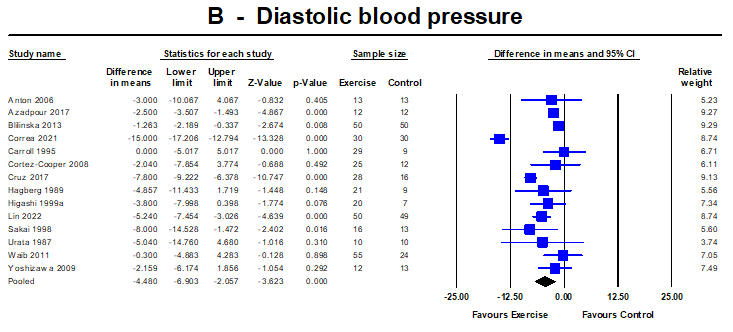  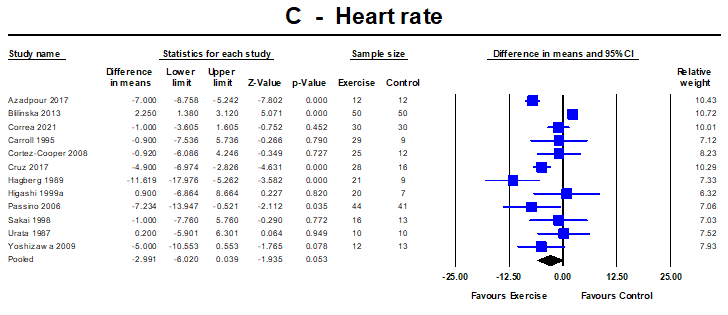 |  |
| --- | --- |

**Supplementary Figure S1: Changes in blood pressure and heart rate**

*Forest plots showing the effects of exercise training on systolic BP [mmHg] (A), diastolic BP [mmHg] (B) and HR [bpm] (C) compared with control. A p-value < 0.05 represents a significant pooled difference in means of overall effect. Horizontal lines across each present 95% CI for each study. The diamond represents the 95% CI for pooled estimates of effect of mean difference.*

| 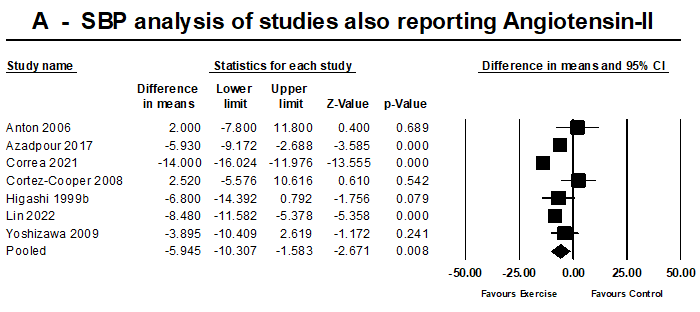 | 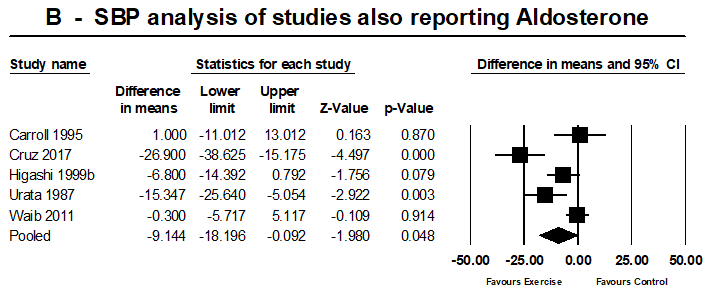 |
| --- | --- |
| 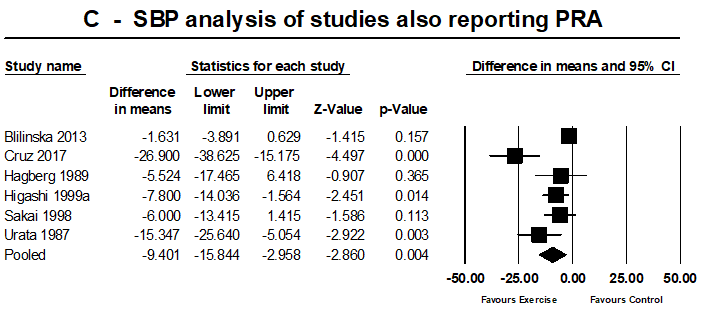 | 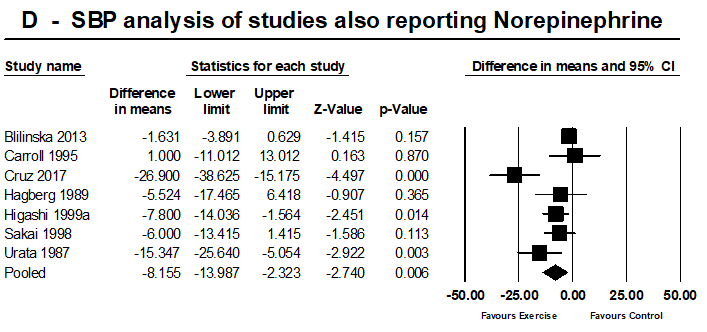 |
| 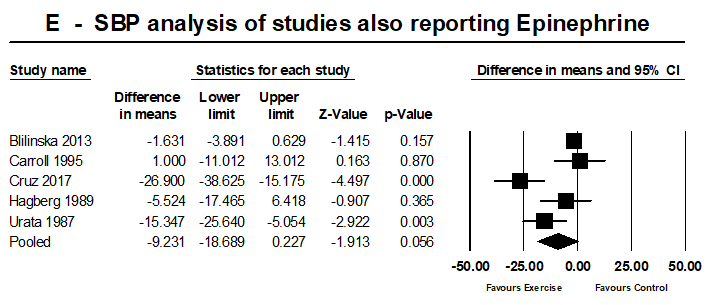 | 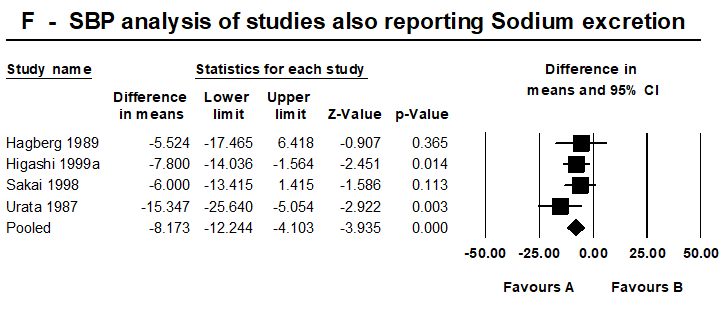 |
| 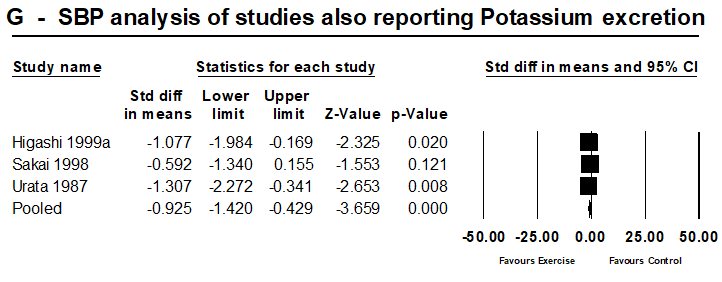 |  |

**Supplementary Figure S2:** **Analysis of net changes in SBP for studies also reporting individual RAAS parameters –**angiotensin II (A), aldosterone (B), plasma renin activity (PRA) (C), norepinephrine (D), epinephrine (E), sodium (F) and potassium excretion (G)

| 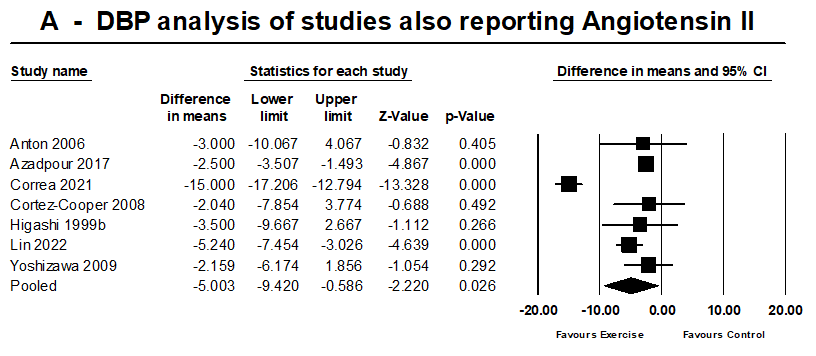 | 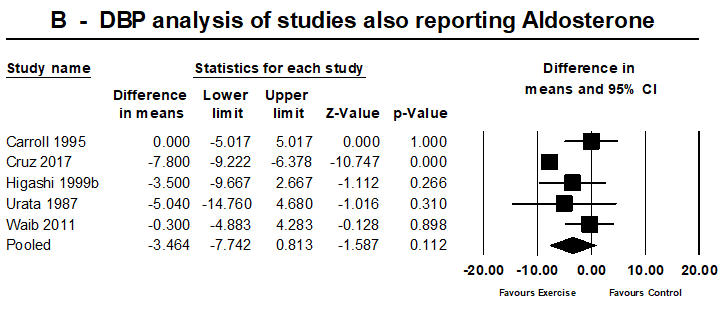 |
| --- | --- |
| 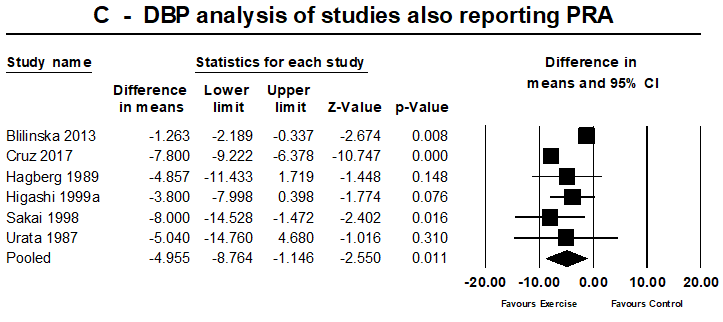 | 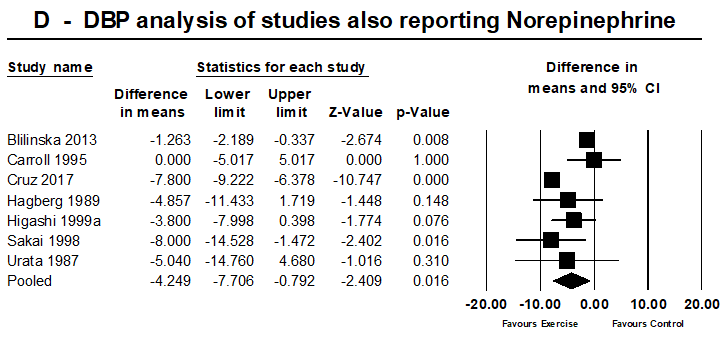 |
| 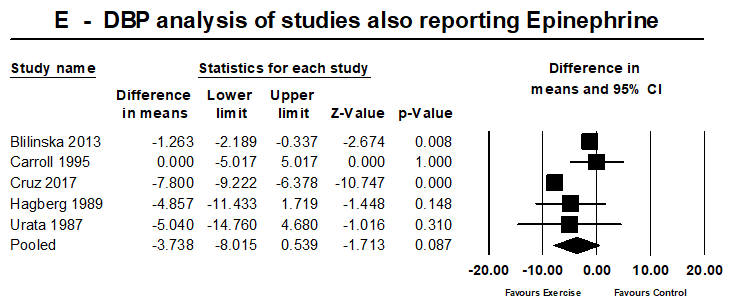 | 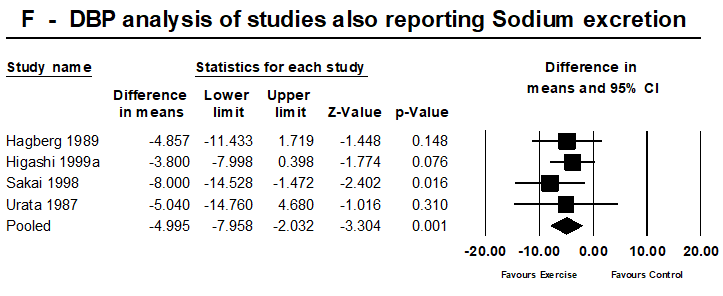 |
| 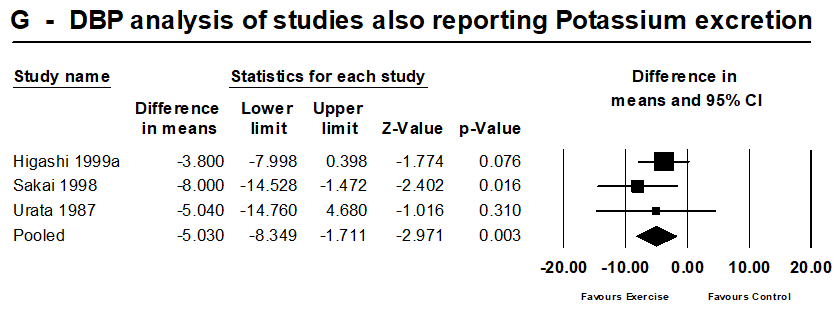 |  |

**Supplementary Figure S3:** **Analysis of net changes in DBP for studies also reporting individual RAAS parameters –**angiotensin-II (A), aldosterone (B), plasma renin activity (PRA) (C), norepinephrine (D), epinephrine (E), sodium (F) and potassium excretion (G)

| 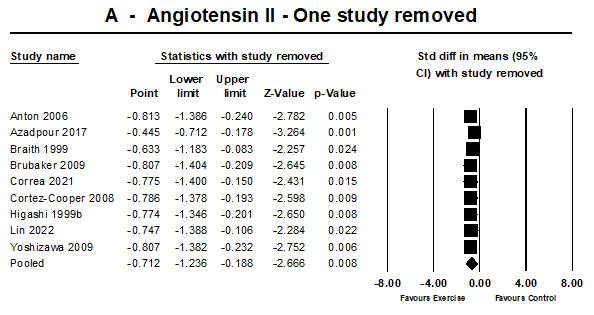 | 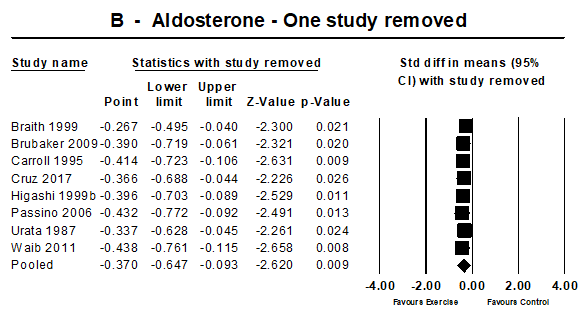 |
| --- | --- |
| 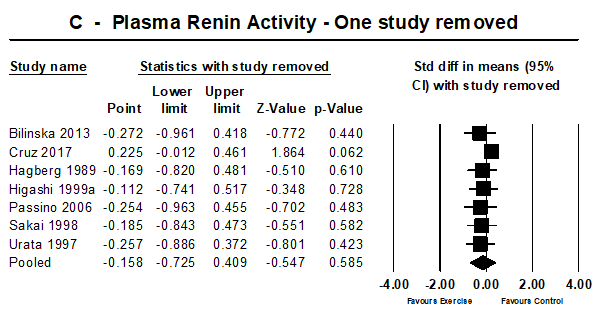 |  |
| 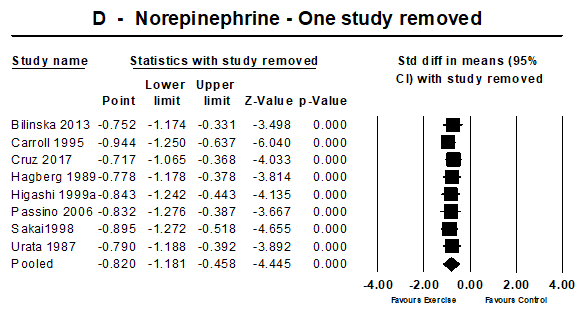  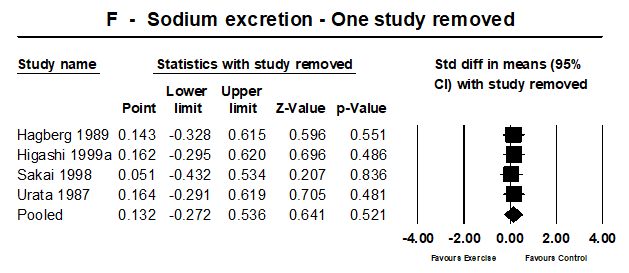 | 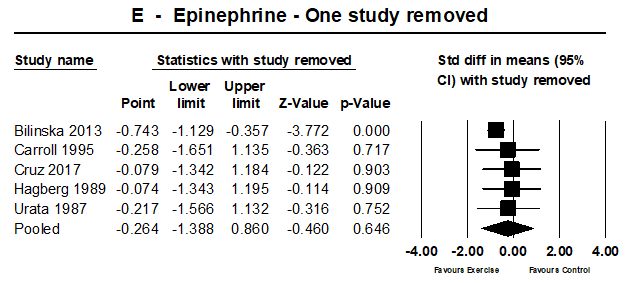  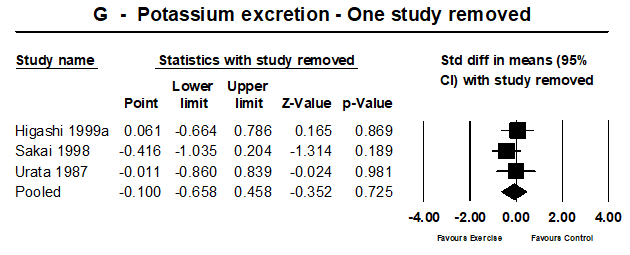 |
| 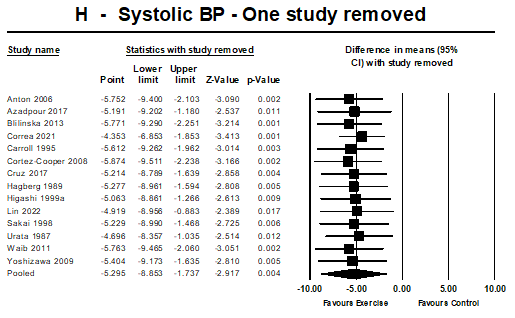 | 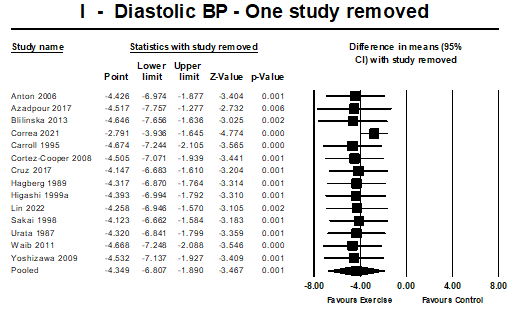 |

**Supplementary Figure S4:** **Sensitivity analyses of changes in RAAS parameters and blood pressure –** angiotensin-II (A), aldosterone (B), plasma renin activity (PRA) (C), norepinephrine (D), epinephrine (E), sodium (F) and potassium (G) excretion, systolic (H) and diastolic (I) blood pressure

| **A** |  | **B** |  |
| --- | --- | --- | --- |
| **C** |  | **D** |  |
| **E** |  |  |  |

**Supplementary Figure S5: Sub-analyses of changes in angiotensin-II**

| **A** |  | **B** |  |
| --- | --- | --- | --- |
| **C** |  | **D** |  |
| **E** |  |  |  |

**Supplementary Figure S6: Sub-analyses of changes in aldosterone**

| **A** |  | **B** |  |
| --- | --- | --- | --- |
| **C** |  | **D** |  |

**Supplementary Figure S7: Sub-analyses of changes in norepinephrine**

| **A** | 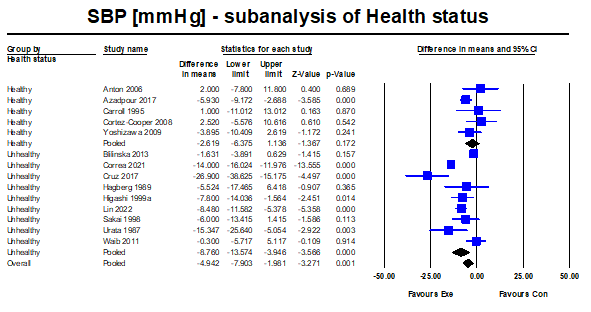 | **B** | 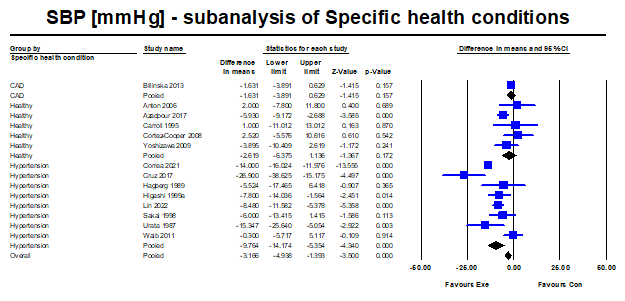 |
| --- | --- | --- | --- |
| **C** | 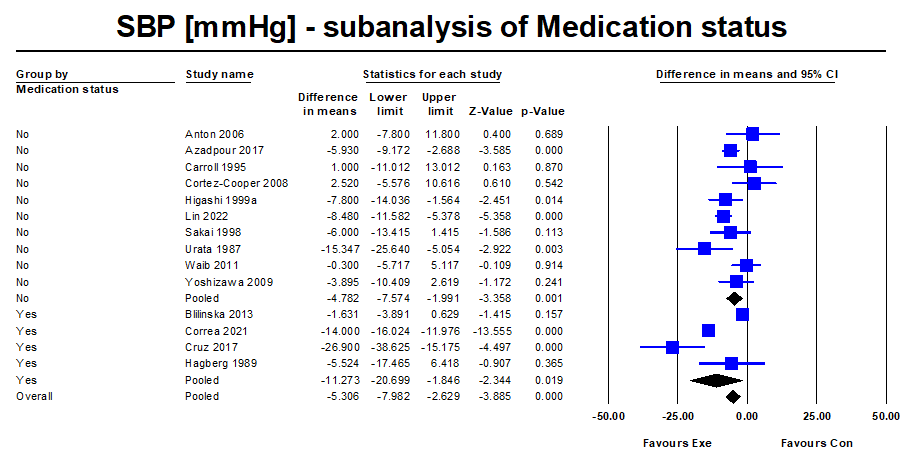 | **D** | 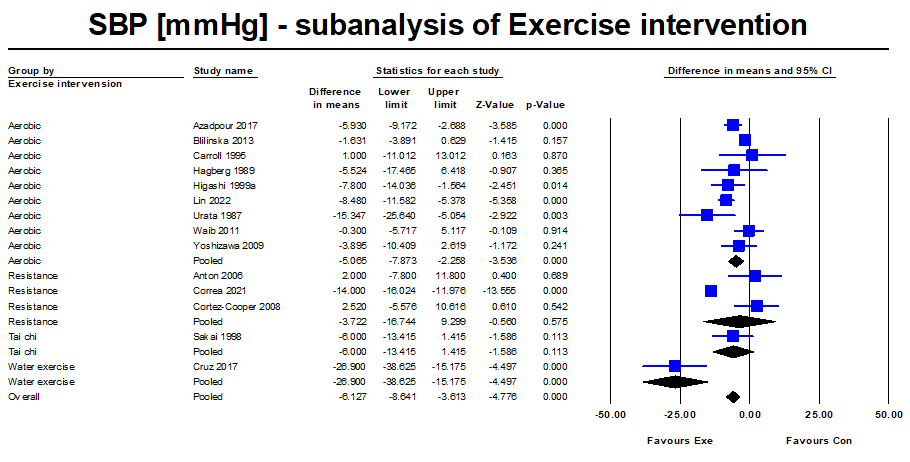 |
| **E** | 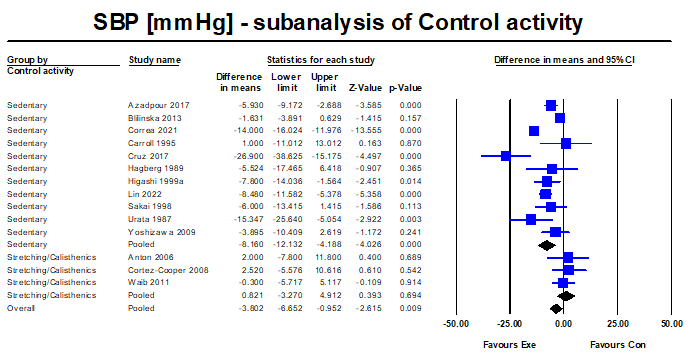 |  |  |

**Supplementary Figure S8: Sub-analyses of changes in systolic blood pressure**

| **A** | 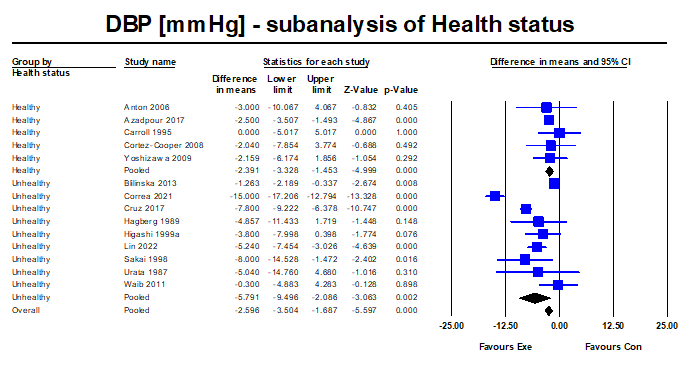 | **B** | 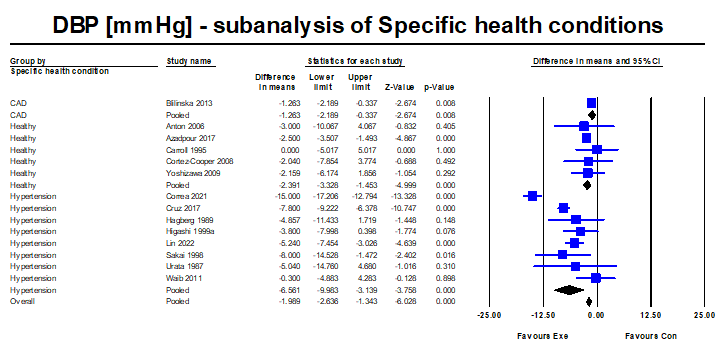 |
| --- | --- | --- | --- |
| **C** | 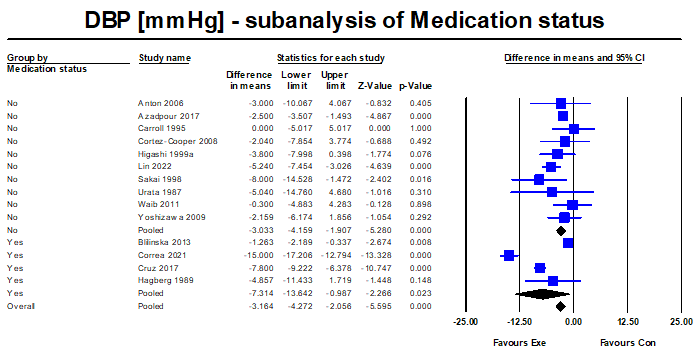 | **D** | 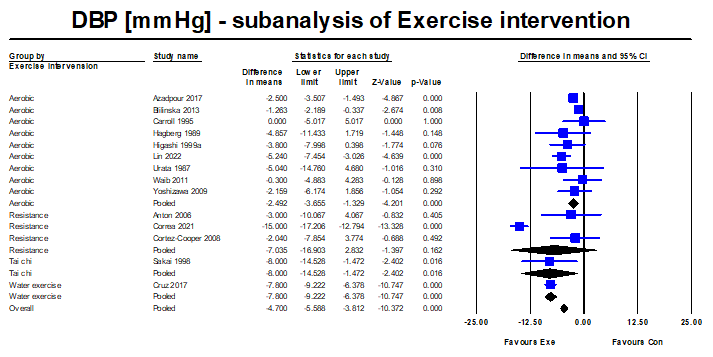 |
| **E** | 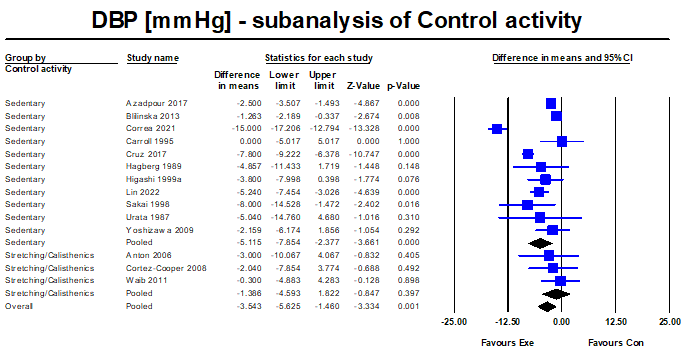 |  |  |

**Supplementary Figure S9: Sub-analyses of changes in diastolic blood pressure**

| A |  | B |  |
| --- | --- | --- | --- |
|  | Plasma renin activity: intercept = -3.44(95% Cl; -10.22 to 3.34), *p* = 0.249 |  | Angiotensin-II: intercept = -2.59(95%CI; -7.4 to 2.26), *p* = 0.247 |
| C |  |  |  |
|  | Aldosterone: intercept = -2.38(95%CI; -5.2 to 0.4), *p* = 0.084 |  |  |
| D |  | E |  |
|  | Epinephrine: intercept = -10.2 (95%CI; -21.67 to 1.27), *p* = 0.066 |  | Norepinephrine: intercept = 1.02 (95%CI; -3.58 to 5.61), *p* = 0.608 |
| F |  | G |  |
|  | Potassium excretion: intercept = -11.33 (95%CI; -39.57 to 16.92), *p* = 0.123 |  | Sodium excretion: intercept = -3.99 (95%CI; -9.25 to 1.27), *p* = 0.082 |

**Supplementary Figure S10: Funnel plots for all parameters –** Plasma renin activity (A), angiotensin-II (B), aldosterone (C), epinephrine (D), norepinephrine (E), potassium (F) and sodium (G)

**References of included studies**

1. Anton MM, Cortez-Cooper MY, DeVan AE, Neidre DB, Cook JN, Tanaka H. Resistance training increases basal limb blood flow and vascular conductance in aging humans. J Appl Physiol 2006;101(5):1351-5.

2. Azadpour N, Tartibian B, Koşar Ş N. Effects of aerobic exercise training on ACE and ADRB2 gene expression, plasma angiotensin II level, and flow-mediated dilation: a study on obese postmenopausal women with prehypertension. Menopause. 2017;24(3):269-77.

3. Bilińska M, Kosydar-Piechna M, Mikulski T, Piotrowicz E, Gąsiorowska A, Piotrowski W, et al. Influence of aerobic training on neurohormonal and hemodynamic responses to head-up tilt test and on autonomic nervous activity at rest and after exercise in patients after bypass surgery. Cardiol J. 2013;20(1):17-24.

4. Braith RW, Welsch MA, Feigenbaum MS, Kluess HA, Pepine CJ. Neuroendocrine activation in heart failure is modified by endurance exercise training. J Am Coll Cardiol. 1999;34(4):1170-5.

5. Brubaker PH, Moore JB, Stewart KP, Wesley DJ, Kitzman DW. Endurance exercise training in older patients with heart failure: results from a randomized, controlled, single-blind trial. J Am Geriatr Soc. 2009;57(11):1982-9.

6. Carroll JF, Convertino VA, Wood CE, Graves JE, Lowenthal DT, Pollock ML. Effect of training on blood volume and plasma hormone concentrations in the elderly. Med Sci Sports Exerc. 1995;27(1):79-84.

7. Corrêa HL, Neves RVP, Deus LA, Maia BCH, Maya AT, Tzanno-Martins C, et al. Low-load resistance training with blood flow restriction prevent renal function decline: The role of the redox balance, angiotensin 1–7 and vasopressin✰,✰✰. Physiol Behav. 2021;230:113295.

8. Cortez-Cooper MY, Anton MM, Devan AE, Neidre DB, Cook JN, Tanaka H. The effects of strength training on central arterial compliance in middle-aged and older adults. Eur J Cardiovasc Prev Rehabil. 2008;15(2):149-55.

9. Cruz LG, Bocchi EA, Grassi G, Guimaraes GV. Neurohumoral and endothelial responses to heated water-based exercise in resistant hypertensive patients. Circ J. 2017;81(3):339-45.

10. Hagberg JM, Montain SJ, Martin WH, 3rd, Ehsani AA. Effect of exercise training in 60- to 69-year-old persons with essential hypertension. Am J Cardiol. 1989;64(5):348-53.

11. Higashi Y, Sasaki S, Sasaki N, Nakagawa K, Ueda T, Yoshimizu A, et al. Daily aerobic exercise improves reactive hyperemia in patients with essential hypertension. Hypertension (Dallas, Tex 1979). 1999;33(1S Suppl):591-7.

12. Higashi Y, Sasaki S, Kurisu S, Yoshimizu A, Sasaki N, Matsuura H, et al. Regular aerobic exercise augments endothelium-dependent vascular relaxation in normotensive as well as hypertensive subjects. Circulation. 1999;100(11):1194-202.

13. Lin B, Jin Q, Liu C, Zhao W, Ji R. Effect and mechanism of tai chi on blood pressure of patients with essential hypertension: a randomized controlled study. J Sports Med Phys Fitness. 2022;62(9):1272-7.

14. Passino C, Severino S, Poletti R, Piepoli MF, Mammini C, Clerico A, et al. Aerobic training decreases B-type natriuretic peptide expression and adrenergic activation in patients with heart failure. J Am Coll Cardiol. 2006;47(9):1835-9.

15. Sakai T, Ideishi M, Miura S, Maeda H, Tashiro E, Koga M, et al. Mild exercise activates renal dopamine system in mild hypertensives. J Hum Hypertens. 1998;12(6):355-62.

16. Urata H, Tanabe Y, Kiyonaga A, Ikeda M, Tanaka H, Shindo M, et al. Antihypertensive and volume-depleting effects of mild exercise on essential hypertension. Hypertension (Dallas, Tex : 1979). 1987;9(3):245-52.

17. Waib PH, Gonçalves MI, Barrile SR. Improvements in insulin sensitivity and muscle blood flow in aerobic-trained overweight-obese hypertensive patients are not associated with ambulatory blood pressure. J Clin Hypertens (Greenwich). 2011;13(2):89-96.

18. Yoshizawa M, Maeda S, Miyaki A, Misono M, Choi Y, Shimojo N, et al. Additive beneficial effects of lactotripeptides and aerobic exercise on arterial compliance in postmenopausal women. Am J Physiol Heart Circ Physiol. 2009;297(5):H1899-903.
